# Modifying and parameterizing the individual-based model inSTREAM for Atlantic salmon and brown trout in the regulated Gullspång River, Sweden

**DOI:** 10.1016/j.mex.2023.102243

**Published:** 2023-06-03

**Authors:** Kristine Lund Bjørnås, Steven Railsback, John Piccolo

**Affiliations:** 1 Department of Environmental and Life Sciences, River Ecology and Management Research Group (RivEM), Karlstad University, Sweden; 2 Norwegian Institute for Nature Research, Trondheim, Norway; 3 Department of Mathematics, Cal Poly Humboldt and Lang, Railsback and Associates, California, USA

**Keywords:** Ecohydraulics, Conservation, Ecological modelling, Water Framework Directive, Salmo salar, Salmo trutta, Environmental flow, Minimum flow, Natural flow regime, Habitat selection, inSTREAM version 6.1 adapted for Atlantic salmon and anadromous brown trout

## Abstract

We modified, parameterized, and applied the individual-based model inSTREAM version 6.1 for lake-migrating populations of landlocked Atlantic salmon (Salmo salar) and brown trout (S. trutta) in a residual flow stretch of the hydropower-regulated Gullspång River, Sweden. This model description is structured according to the TRACE model description framework. Our aim was to model responses in salmonid recruitment to alternative scenarios of flow release and other environmental alterations. The main response variable was the number of large out-migrating juvenile fish per year, with the assumption that individuals are more inclined to out-migrate the larger they get, and that migration is an obligatory strategy. Population and species-specific parameters were set based on local electrofishing surveys, redd surveys, physical habitat surveys, broodstock data as well as scientific literature.

• Simulations were set to run over 10 years, with sub-daily time steps, in this spatially and temporally explicit model.
• Model calibration and validation of fish growth was done using data on juvenile fish from electrofishing.
• The results were found to be sensitive to parameter values for aggregated fish, i.e., “superindividuals” and for the high temperature limit to spawning.

## Graphical abstract

**Figure:**
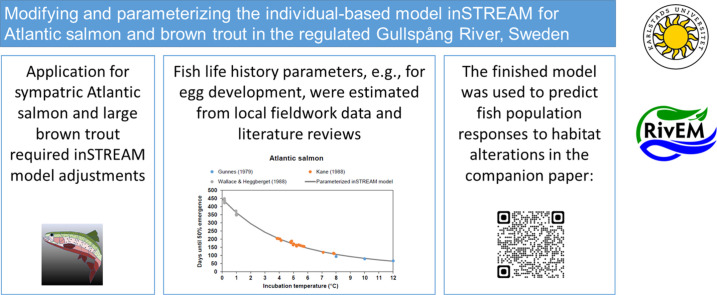

Specifications table

**Table:** 

Subject area:	Agricultural and Biological Sciences
More specific subject area:	Ecohydraulics
Name of your method:	inSTREAM version 6.1 adapted for Atlantic salmon and anadromous brown trout
Name and reference of original method:	Railsback, S.F., Harvey, B.C., Sheppard, C., 2014. inSTREAM-SD: The individual-based stream trout research and environmental assessment model with sub-daily time step, version 6.1. Lang, Railsback & Associates, Arcata, California, USA. Available at: https://ecomodel.humboldt.edu/sites/default/files/ecomodel/instream_6-1_modeldescription.pdf
Resource availability:	https://ecomodel.humboldt.edu/instream-insalmo-recent-2012-2020-versions

## Method details

This document provides supporting evidence that our model was thoughtfully designed, correctly implemented, thoroughly tested, well understood, and appropriately used for its intended purpose. This method description is written in the TRACE (“TRAnsparent and Comprehensive model Evaludation”) model-reporting format, following the rationale of Schmolke et al. [77] and using the standard terminology and document structure in Grimm et al. [33] and Augusiak et al. [4]. The eight TRACE elements are: (1) Problem formulation, (2) Model description, (3) Data evaluation, (4) Conceptual model evaluation, (5) Implementation verification, (6) Model output verification, (7) Model analysis, and (8) Model output corroboration. We here document the context and research problems to which the model was applied in the companion paper Bjørnås et al. [9]. This includes revisions and adjustments of the inSTREAM code, parameterization for our local study site and species, and validation and tests of the final model.

## Problem formulation

In this TRACE element we describe the decision-making context in which we applied the individual-based Stream Trout Research and Environmental Assessment Model (henceforth referred to as inSTREAM).

### The Gullspång River

**Fig. 1:**
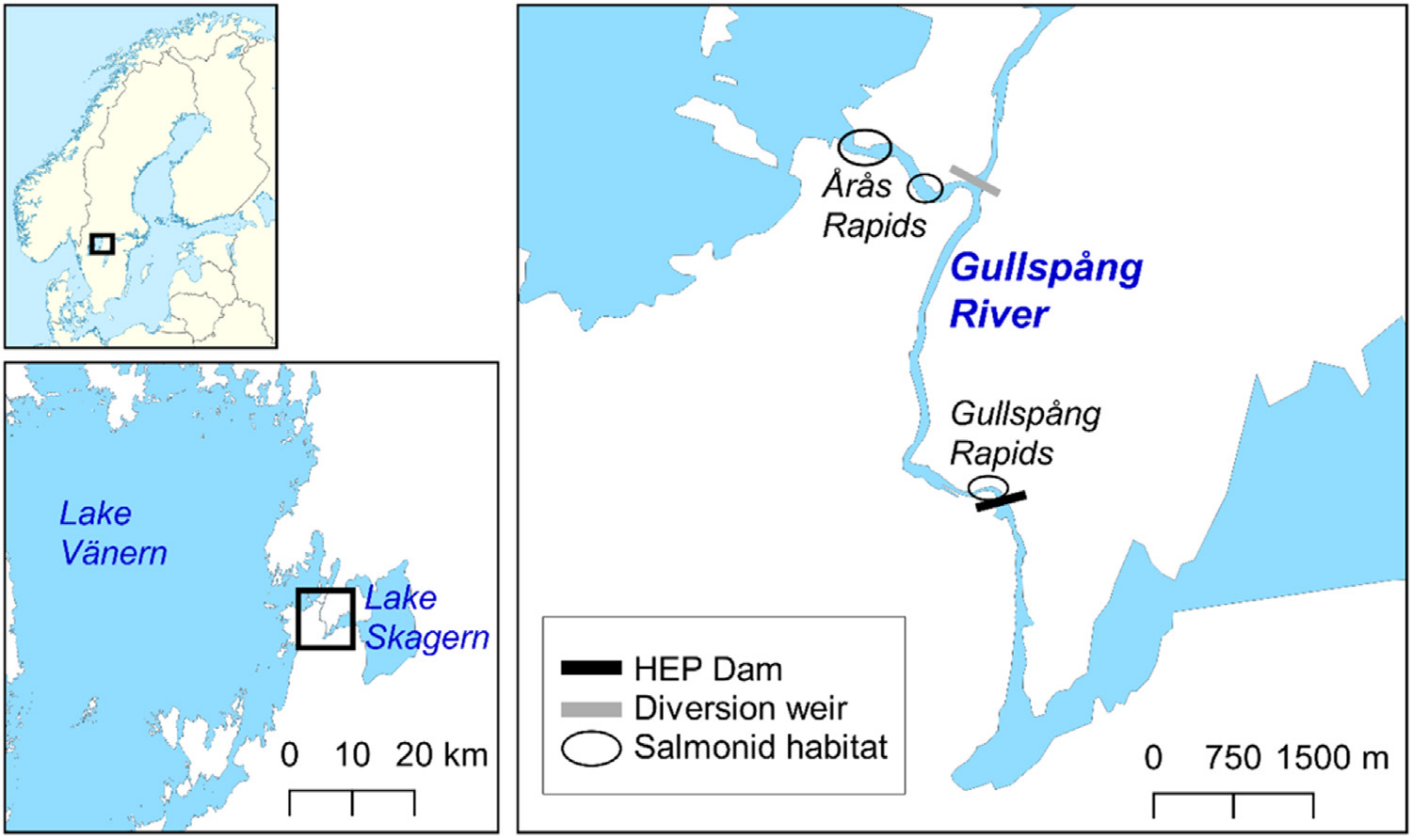
The Gullspång River drains Lake Skagern to Lake Vänern, Southern Sweden. Our study site, the Gullspång Rapids, is the most upstream of the three salmonid spawning and rearing areas in the river (circled).

We modified the spatially explicit individual-based salmonid model inSTREAM-SD version 6.1 for the Gullspång rapids (hereafter G-Rapids), a residual flow reach in the Gullspång River, Sweden (Fig. 1).

Previous to construction of the Gullspång hydroelectric power plant (HEP) dam in in 1906-1908, the G-Rapids was the main river channel and one of three reaches with Atlantic salmon (*Salmo salar*) and brown trout (*Salmo trutta*) spawning and nursery habitat in the Gullspång River. The local Gullspång River populations of landlocked Atlantic salmon and brown trout are of high conservation value [41,59]. The Gullspång salmon became landlocked with the postglacial uplift, and now migrate between Lake Vänern and the roughly 6 km long Gullspång River. After the river was impounded and water diverted to the HEP, the G-Rapids were lost as salmon and trout habitat for about a century, leaving the two species to spawn and rear only in the Small and Large Årås Rapids (Fig. 1; [54]).

In the early 2000s, sanctioning of a minimum flow and extensive restoration measures transformed the G-Rapids back into a somewhat functioning salmonid habitat. Now the area generally has the highest juvenile salmon and trout densities in the whole river [30,78]. However, continuous remedial measures are necessary, as the dam disturbs natural river processes such as erosion and sediment transport. Consequently, river managers want to further improve the salmonid habitat of this reach.

The goal of the model parameterization and application was to address the potential for additional habitat remedial measures and environmental flow release to increase production of Atlantic salmon and brown trout in the G-Rapids. Unless otherwise is specified, “salmon” and “trout” henceforth refer to Atlantic salmon and brown trout.

### Relicensing and environmental flows in Sweden and in the EU

The modeling work has high societal relevance and timeliness in a wider geographical context. As part of the Swedish implementation and interpretation of the EU Water Framework Directive, all Swedish hydropower schemes will go through a 20-year relicensing process of to be in alignment with national environmental legislation. Therefore, there is a need for tools that predict the ecological effects of different flow regimes for important species like Atlantic salmon and brown trout in a realistic and transferrable way.

### Stakeholders

Stakeholders for the modeling project are the local and regional environmental management agencies in Sweden, hydropower companies (mainly Fortum Sweden, as owner the hydropower rights in the Gullspång River), and the local communities for which the salmon is important for local identity and tourism. Other involved management groups include the Gullspång River Action Plan (GRAP), Gullspångsälvens Vattenvårdsförbund (the Gullspång River Catchment's water management group), Förvaltning Vänerlax (Management group for Vänern salmon), and the Natura 2000 Protected Area managers.

### Stakeholder contributions

Fortum AB funded the physical habitat surveys used for the hydraulic modeling. Officials from Mariestad, Töreboda and Gullspång municipalities provided valuable local knowledge and help with field data collection. Jukka Syrjänen, manager of the Gullspång River redd surveys, and Johnny Norrgård, Gammelkroppa hatchery, both contributed greatly with information from hatchery protocols and rich local knowledge. The electrofishing management team cooperated to collect length-weight data on sedated juveniles from the river. The Gullspång River Action Plan (GRAP) participated with ideas and discussion about experimental scenarios.

### Research questions

#### What are the effects of increasing the minimum flow?

A minimum flow to maintain ecological integrity of river ecosystems is often referred to as ‘environmental flow’ [32,62], although the concept of environmental flows includes an entire flow regime in terms of quantity, quality and timing of water flows that balance ecological needs with societal needs (The Brisbane [81]). Prescribing an environmental flow regime, rather than a minimum level, is however more complex than the scope of our modeling project.

Current minimum flow requirements for the G- Rapids of 3 m^3^/s year-round come from the latest verdict [18]. However, the 3 m^3^/s requirement is waived if water levels in Lake Skagern drop below 68.5 m a.s.l. An increase in mandated minimum flow to the G-Rapids incurs a *cost* for the hydropower company in terms of water lost for production (Table 1), which can be contrasted with predicted salmon and trout production as *benefit*. We constructed four minimum flow-based scenarios (Table 1). Detailed input files are described in section ``Reach discharge''.

**Table 1:** Summary of all flow scenarios used in flow experiments.

Scenario	Description	Mean volume (GL/year)
Flow0	Near actual flow; null scenario	126.3
Flow3	Strict enforcement of 3 m^3^/s minimum flow	127.9
Flow6	Constant minimum flow 6 m^3^/s	205.6
Flow9	Constant minimum flow 9 m^3^/s	291.1
SHYPE1	10% of total natural daily discharge	197.8
SHYPE2	20% of total natural daily discharge	387.8
SHYPE3	33% of total natural daily discharge	567.0

#### What are the effects of a more natural flow regime?

**Fig. 2:**
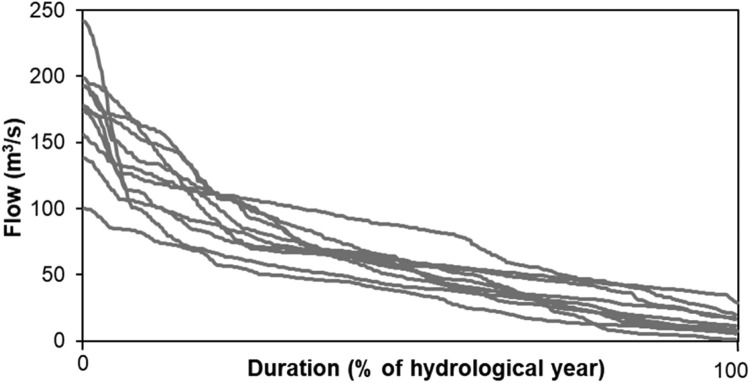
Flow duration curves (grey lines) from S-HYPE [79] over the hydrological years of our modeling project. A hydrological year is set as 20 September to 19 September the following calendar year.

The natural flow paradigm, coined by Poff et al. [61], has been highly influential in the development of environmental flows science [60]. Restoring the natural flow regime has been regarded the holy grail in river restoration [61], although reference flow conditions are difficult to assess as anthropogenic disturbances of rivers and streams are globally pervasive (e.g., [19]) and date far back in time. To contrast the effect of a highly artificial year-round stable flow with a more naturally dynamic flow, we made three natural flow-based scenarios (Table 1) based on hydrologically modelled flow (S-HYPE by SMHI [79]) (Fig. 2).

#### What are the effects of increasing the number of returning spawners?

If flow restoration leads to increased juvenile production, then more adults will eventually return to the G-Rapids to spawn. However, juvenile production can only increase until carrying capacity is reached. Density dependent mortality in early salmonid life-stages is well documented (e.g., [52]). We investigated the stock-recruitment relationship of the reach by making scenarios with different spawner densities under the same physical environmental conditions.

### Key model output

The yearly number of out-migrating fish equal to or above 12 cm (hereafter referred to as “large out-migrants”) is our main model output. This version of inSTREAM reports the number of and average length of out-migrating fish on every time step, therefore the criteria ≥ 12 cm refers to mean length of one or several fish. The length criterium was added because we assume that smaller (< 12 cm) out-migrants have lower survival during out-migration and in the lake. The timing of out-migration and exact size of out-migrants comes from the out-migration method in which individuals are more likely to out-migrate the larger they are (section ``Outmigration''; section ``Out migration''). The number of large out-migrants serves as an index of juvenile retention, growth, and survival in the G-Rapids.

### Domain of applicability

Models in the inSTREAM/inSALMO family have been used for a range of applications and salmonid species (e.g., brown trout: [5,37], rainbow/steelhead trout: [40], and Chinook salmon: [20]). Our model implementation is an explicit description of the conditions of the G-Rapids during 20 September 2008 - 20 September 2018 for local trout and salmon, but the method can also be used in other systems with anadromous or lake-migrating salmonids. Some of our fish parameters are site-specific and/or species-specific. For these, the parameterization process is explained in detail under Data evaluation.

## Model description

This TRACE element provides supporting information about the inSTREAM 6.1 model itself. Normally, TRACE documents for individual-based models include a full model description in a format that follows the ODD protocol [34,35]. However, the model used here is modified from an inSTREAM version (6.1) that has already been comprehensively and systematically described in Railsback et al. [72]. This model description is not organized in the ODD format, but instead follows the layout of the original, full description of inSTREAM 6.1. We describe only the changes we made and suggest that the original model description is read alongside for reference (“MD” refers to the original model description, Railsback et al. [72]).

### Introduction, Objectives, and Background (MD: 1.)

#### Purpose and overview (MD: 1.2.)

The purpose of our application is to evaluate emergent population responses of Atlantic salmon and brown trout from simulated flow regulation changes in the residual flow reach G-rapids.

#### Summary of changes from version 6.1 (MD: 1.2.)

In this application, all fish are obligate anadromous and semelparous. Arriving spawners are added to the model over a period in the fall of every model year, using what corresponds to the stocking input files in the MD. All surviving juvenile fish eventually migrate out of the model area based on an outmigration probability that increases with increasing length. In this application, habitat cells are hexagonal and uniformly sized. The superindividual concept, where several young of the year fish are represented in an aggregated form, is borrowed from inSALMO for computational efficiency. Temperature is input as hourly temperature values.

### Overview of inSTREAM-SD (MD: 2.)

#### Fundamental assumptions (MD: 2.1.)

The only change from the MDs fundamental assumptions is that the habitat input variable temperature is input on hourly steps in our version.

#### Summary of model actions and schedule (MD: 2.3.)

1) Model:
  a) Update simulation time, day/night phase.
2) Reaches:
  a) Input hourly flow, drift food concentration and temperature.
  b) If time = 00 h, input daily turbidity.
3) Fish (if date = 1 January and time = 00 h):
  a) Increment age by 1 year.
  b) Divide superindividual object into corresponding number of fish
4) Reaches (if time = 00 h):
  a) Update maximum fish lengths
5) Model (if time = 10 h on a day when arrival occurs):
  a) Arrival of adults
6) Fish (if time = first daytime hour):
  a) Spawn
7) Redds (if time = first daytime hour):
  a) Survive
    I Dewatering
    II Scouring
    III Low temperature
    IV High temperature
    V Superimposition
  b) Develop
  c) Emerge
8) Model:
  a) Determine whether a new time step begins due to change in flow, temperature or day/night phase.
9) Fish (if step occurs this hour):
  a) Survive
    I) High temperature
    II) High velocity
    III) Stranding
    IV) Aquatic predation
    V) Terrestrial predation
    VI) Poor condition
    VII) Outmigration
  b) Grow
10) Model (if step occurs this hour):
  a) Report output for ending time step
11) Fish (if step occurs this hour):
  a) Select habitat and activity

### Habitat Variables and Methods (MD: 4.)

#### Reaches (MD: 4.1.)

##### Reach-scale variables (MD: 4.1.1.)

*Time-series inputs:* Reach temperature is input with hourly values, so that temperature is updated on each model time step. Many processes in inSTREAM are temperature-dependent and were designed to use daily means as the temperature variable. To capture potential effects of sub-daily temperature variation, we adjusted some temperature-dependent processes to use hourly temperature input. Temperature effects on incubating eggs (both mortality and development time) were assumed to remain adequately represented by daily mean temperature. This assumption was made in part because simulated eggs, unlike fish, do not have adaptive behaviors that depend on temperature, and in part because eggs are buried in redds, which likely damps within-day temperature variation. Temperature effects on simulated fish growth, survival, and behavior were changed to use hourly temperature input. Each site's internal variable for current temperature is updated on any hour in which changes in either flow or drift food concentration, or the change between day and night, triggers a model time step (Schedule 2.2.2. step 8A). Whether or not the temperature criterium for spawning is met is determined using the hourly temperature at the first hour of daylight. Fish behaviors are likely to be more variable within a day with this revised model formulation. We could however not attain hourly temperature data, so daily mean temperatures were instead used as the hourly input.

*Piscivorous fish density*: the piscivorous trout formulation is built in to inSTREAM 6.1. However, we assume no predation by other fish in the model system because 1) salmon and brown trout migrate out of the reach before they reach a size where they become fish-eating, 2) adult salmonid fish do not eat while in the river to spawn, and 3) salmon and trout are the only species that can migrate up the fish ladder. We have therefore chosen parameter values (section ``Density of predatory trout is zero''.) so that the piscivorous trout and salmon density always remains zero.

### Fish Variables and Methods (MD: 5.)

In this version, feeding methods vary between juveniles and adults, hence we added a Boolean state variable for whether a fish is an adult, *isSalmonSpawne*r. In addition, the fish state variable *nRep* was added to allow for superindividual representation of young of the year fish (section ``New fish attributes''), where the *nRep* stated the number of fish individuals the fish represents. The value of *nRep* is always one for fish aged 1+ and older, and for fish in the initial population (section ``Fish Initialization'').

#### Spawning (MD: 5.1.)

This version does not include the full life cycle of salmon and trout.

##### Decide whether to spawn (MD: 5.1.1.)

Whether or not the temperature criterium for spawning is met is determined using the hourly temperature at the first hour of daylight.

##### Select spawning cell and move there (MD: 5.1.2.)

We made changes to the spawning cell selection submodel to avoid unrealistic redd superimposition due to stable flow conditions. Spawning females still select the cell with the highest *spawnQuality,* but we added a stochastic term. With the original inSTREAM 6.1 submodel, equal *spawnQuality* occurred when several cells had maximum suitability (=1) of velocity and of depth, as well as spawning gravel coverage (because the cells were uniformly sized). When several cells had the same *spawnQuality* the cell with the lowest *CellID* number was chosen as the spawning cell by the model. To avoid this problem, we added a small error term, *U*, to the *spawnQuality* formula and introduced a new parameter, *fishSpawnCellUncert*, to control the range of *U*. The error *U* is calculated for each cell by each potential spawner on a time step as 1 + *fishSpawnCellUncert* times a random number between zero and one. *SpawnQuality* is thereby calculated as: spawnQuality=spawnDepthSuit×spawnVelocitySuit×spawnGravelArea×U

##### Incur weight loss (MD: 5.1.5.)

In the original inSTREAM version 6.1, fish can be repeat spawners. In this version, salmon and trout die after spawning. This is modeled by 1) having adult fish always receive zero food intake and 2) incurring a significant weight loss after spawning. In addition, a %- weight loss was incurred on each time step of the last day of the spawning period for adult fish that for some reason did not spawn within the spawning period. This makes also these fish starve and die due to poor condition within a short time.

#### Habitat and activity selection (MD: 5.2.)

##### Overview (MD: 5.2.1.)

As adults’ food intake is zero (section ``Overview (MD: 5.3.1.)''), their habitat selection (except in the time step when females choose spawning cell) will consist of choosing cells that provide a good tradeoff between low predation risk and low swimming cost.

##### Competition via dominance hierarchy (MD: 5.2.1.)

All fish, regardless of species, are part of the same length-based dominance hierarchy.

##### Identification of potential destination cells (MD: 5.2.2.)

*Distance limitation*: A fish's potential destination cells always include cells adjacent to the fish's current cell. With hexagonal cells, there are six adjacent cells available to small fish even if they are further away than the *maxMoveDistance* (MD: 5.2.2.1).

*Effects of length*: A fish's length affects expected fitness. InSTREAM assumes Fish select their activity (feeding vs. hiding) and habitat cell by maximizing the *expectedMaturity,* a term that represents their expected probability of surviving both predation and starvation over a future time horizon. In inSTREAM 6.1, the fitness measure also includes a term encouraging fish to grow, because otherwise simulated fish tend to feed no more than necessary to maintain their current length and weight. Preliminary simulations indicated that the growth term was not resulting in sufficient growth, so it was replaced with a simpler and stronger term. The revised growth term is ln(1.0+expectedLength/biggestLength) where *expectedLength* is the length the fish would be at the end of the decision time horizon and *biggestLength* is the greater of (a) the length of the largest fish (of any of the included species) currently in the simulation and (b) 1.5 times the minimum spawning length for the fish's species. This term encourages fish of all sizes to continue growing, but the value of growth decreases (due to the logarithm) as length increases (MD: 5.2.5.2).

#### Feeding and Growth (MD: 5.3.)

##### Overview (MD: 5.3.1.)

Adult salmon and trout do not feed in the river when they return to spawn. In our application, this is represented in the food intake calculations: drift food and search food intake is zero in all cells for adult fish, i.e., adult fish cannot find food in any cell.

##### Food intake: drift feeding strategy (MD: 5.3.3.)

The model parameter *fishDetectDistNightFactor* is called *fishReactDistNightFactor* in this version.

#### Fish survival (MD: 5.4.)

Mortality due to angling is not applicable to this system, thus all angling parameters are turned off.

##### High temperature (MD: 5.4.1.)

As with other mortality factors, inSTREAM 6.1 calculates the daily survival from high temperature on each time step and adjusts survival for the length of the time step. As temperature is entered per hour in this application, temperature survival is calculated from the reach temperature on the current time step. Note that this is an approximation because temperature mortality is likely to be time-scale dependent, e.g., fish might tolerate 26°C for one hour but have low survival if high temperatures persist over one or more days. For our application, however, this approximation is valid as water temperatures are not extreme and climate change scenarios are not considered in this application.

##### Aquatic predation (MD: 5.4.4.)

In previous versions of inSTREAM, the parameters setting the baseline level of aquatic predation are fish parameters. In our application, fish parameters *fishMortAq-PredDayMin* and *fishMortAqPredDayMin* are replaced with corresponding reach habitat parameters *habFishMortAqPredDayMin and habFishMort-AqPredNightMin*. These parameters represent the daily probability of surviving aquatic predation under conditions where the survival increase functions offer no reduction in risk, under day and night conditions, respectively. As previously mentioned, we expect aquatic predation to be negligible in this system. Thus, parameter values for this submodel are set very high to make it inactive. The turbidity survival increase function is also turned off due to all zero turbidity values. The survival increase parameters *mortFishAqPredF9* and *mortFishAqPredF1* (MD: Table 17) are taken out from our model version.

##### Terrestrial predation (MD: 5.4.5.)

The parameters *mortFishTerrPredF1* and *mortFishTerrPredF9* (MD: Table 18) were removed in the current model version.

##### Outmigration

In our application, out-migration of juvenile salmon and trout is implemented in terms of juveniles becoming more likely to out-migrate as their size increases. The event of a juvenile salmonid migrating downstream is treated as a type of mortality: when a simulated juvenile salmon decides to out-migrate, it is immediately removed from the simulation. Out-migration is modeled as a stochastic event, with the probability of out-migration being a logistic function of fish length. The logistic function is parameterized to reproduce some assumed probabilities of remaining in the reach (i.e., the cumulative probability of not migrating out over all time steps up to the present), at an assumed growth rate of 0.5% increase in length per day. The assumed probabilities in our application are discussed in section ``Out migration''. The outmigration parameters *mortFishOutmigrationL1* and *mortFishOutmigrationL9* (lengths at which daily probability of outmigration is 0.1 and 0.9) are fitted to reproduce assumed cumulative probabilities (section `Out migration'').

##### Angling and hooking (MD: 5.4.7.)

Fishing mortality is not included in our inSTREAM application.

### Redd Variables and Methods (MD: 6.)

#### Emergence (MD: 6.3.)

##### New fish attributes (MD: 6.3.2.)

To improve execution speed, we implemented the superindividual concept from inSALMO [67]. This concept is most appropriate for juvenile fish because they are most numerous and because they consume relatively little food and use little hiding space compared to what is available in each cell. Artifacts of cell size can however become important when each superindividual consumes much or all the food or space in typical cells. Superindividuals were implemented as the following:

• Each fish species has been given a new parameter, the *juveSuperindividualRatio*, which is the value given to *nRep* when simulated fish are created upon emerging from the redd. Previous experience [65] indicated that values of 20 or lower have little effect on results.
• Only new juveniles created via hatching and emerging from redds are created as superindividuals. When each redd is fully developed, the number of simulated fish is set to the number of eggs divided by *juveSuperindividualRatio*, and *nRep* of each such fish is set to *juveSuperindividualRatio*. If uneven, the number of eggs is rounded upwards.
• Superindividuals are divided into regular individuals when they reach age 1, which happens on the first January 1 of their life. When any fish with *nRep* > 1 turns from age 0 to age 1, it creates (*nRep* – 1) more identical individuals that each have *nRep* set to 1.
• When superindividuals consume resources (drift or search food, hiding space), the amount consumed is multiplied by *nRep*.
• Output files are modified to adjust results by the value of *nRep*: the live population and mortality output files report the number of fish represented by superindividuals.

### Initialization (MD: 7.)

#### *Fish Initialization* (MD: 7.4.)

The value of *nRep* for fish initialized at the start of a simulation is one, i.e., each model fish represents one fish individual, regardless of age class.

### Trout stocking (MD: 8.)

In our application, the trout stocking method is modified to represent the natural arrival of adult salmon and trout every year. The arrival/stocking file is otherwise identical. The sex of arriving fish is assigned randomly, with a 50% probability of being female, and their lengths were drawn from the normal distribution defined by the input mean and standard deviation.

## Data evaluation

This TRACE element provides supporting information on the sources of quantitative and qualitative data used to parameterize the model. We also present observed patterns and site-specific data used to parameterize the model.

### Model parameters

This is the first time inSTREAM has been applied for Atlantic salmon, as well as the first time for brown trout with a diadromous life-history strategy. We therefore attained relevant site-specific and reviewed literature in order to best describe these populations and species (Table 2). This section details how we parameterized and prepared input data for inSTREAM 6.1 in the Gullspång Rapids.

**Table 2:** Relevant parameters in our model application. Sp means species-specific parameter values were used.

Parameter	Entity	Submodel/method
fishSpawnMinAge	Fish	Spawning criteria
fishSpawnMinLength	Fish	Spawning criteria
fishSpawnMinCond	Fish	Spawning criteria
fishSpawnStartDate	Fish (Sp)	Spawning criteria
fishSpawnEndDate	Fish (Sp)	Spawning criteria
fishSpawnMinTemp	Fish	Spawning criteria
fishSpawnMaxTemp	Fish	Spawning criteria
habMaxSpawnFlow	Reach	Spawning criteria
fishSpawnMaxFlowChange	Fish	Spawning criteria
fishSpawnCellUncert	Fish	Spawning site selection
fishSpawnDSuitD(1-5)	Fish	Spawning site selection
fishSpawnDSuitS(1-5)	Fish	Spawning site selection
fishSpawnVSuitV(1-6)	Fish	Spawning site selection
fishSpawnVSuitS(1-6)	Fish	Spawning site selection
fishFecundParam(A-B)	Fish (Sp)	Create a redd
fishSpawnEggViability	Fish	Create a redd
fishSpawnWtLossFraction	Fish	Spawning weight loss
fishMoveDistParam(A-B)	Fish	Habitat selection
fishFitnessHorizon	Fish	Expected maturity
fishWeightParam(A-B)	Fish (Sp)	Growth
fishDetectDistanceParam(A-B)	Fish	Drift-foraging
fishReactDistNightFactor	Fish	Drift-foraging
fishCaptureParam(1&9)	Fish	Drift-foraging
habDriftRegenDist	Reach	Drift-foraging
habSearchProd	Reach	Search food foraging
fishSearchArea	Fish	Search food foraging
fishSearchNightFactor	Fish	Search food foraging
fishCmaxParam(A-B)	Fish	Max consumption
fishCmaxTempT(1-7)	Fish	Max consumption
fishCmaxTempF(1-7)	Fish	Max consumption
habShelterSpeedFrac	Reach	Swimming costs
fishRespParam(A-D)	Fish	Respiration costs
habPreyEnergyDensity	Reach	Net energy intake
fishEnergyDensity	Fish	Growth
mortFishHiTT(9&1)	Fish	High temperature mortality
fishMaxSwimParam(A-E)	Fish	Max sustained swim speed
mortFishVelocityV(9&1)	Fish	High velocity mortality
mortFishVelocityCoverFactor	Fish	High velocity mortality
mortFishStrandD(1&9)	Fish	Stranding mortality
habMortFishAqPredNightMin	Reach	Aquatic predation mortality
habMortFishAqPredDayMin	Reach	Aquatic predation mortality
mortFishAqPredCoverFactor	Fish	Aquatic predation mortality
mortFishAqPredD(9&1)	Fish	Aquatic predation mortality
mortFishAqPredL(1&9)	Fish	Aquatic predation mortality
mortFishAqPredT(9&1)	Fish	Aquatic predation mortality
mortFishTerrPredNightMin	Fish	Terrestrial predation mortality
mortFishTerrPredDayMin	Fish	Terrestrial predation mortality
mortFishTerrPredD(1&9)	Fish	Terrestrial predation mortality
mortFishTerrPredV(1&9)	Fish	Terrestrial predation mortality
mortFishTerrPredL(1&9)	Fish	Terrestrial predation mortality
cellDistToHide	Cell	Terrestrial predation mortality
mortFishTerrPredCoverFactor	Fish	Terrestrial predation mortality
mortFishTerrPredH(9&1)	Fish	Terrestrial predation mortality
mortFishConditionK(1&9)	Fish	Poor condition mortality
mortReddDewaterSurv	Redd	Redd survival
mortReddScourDepth	Fish	Redd survival
habShearParam(A&B)	Reach	Redd survival
mortReddLoTT(1&9)	Fish	Redd survival
mortReddHiTT(1&9)	Fish	Redd survival
cellArea	Cell	Redd survival
cellFracSpawn	Cell	Redd survival
reddSize	Redd	Redd survival
reddDevelParam(A-C)	Fish (Sp)	Redd development
reddNewLengthMean	Fish	Creation of new fish
reddNewLengthStdDev	Fish	Creation of new fish
juveSuperindividualRatio	Fish	Creation of new fish

### Main site-specific data sources

#### Ex situ populations in River Klarälven

Salmon and trout of Gullspång River origin have been popular for hatchery rearing and introduction for angling to other freshwater systems because of their fast growth (Johnny Norrgård, pers.comm.) and potential to attain a large size (e.g., [54]). In the River Klarälven, draining to the northern part of Vänern, Gullspång-originating hatchery (hereafter GH) salmon and trout are released for angling, but also to act as a living gene bank for wild Gullspång salmon and trout in the Gullspång River [57]. We therefore refer to the GH salmon and trout as ex situ populations throughout this paper.

GH salmon and trout are incubated and reared in Gammelkroppa lax AB hatchery station and stocked into River Klarälven as part of the hydropower company's compensatory stocking program [59]. As in the Gullspång River, they migrate to Lake Vänern and stay there until maturity. Adult salmon and trout are then captured early in their spawning migration, in a fish trap at the lowermost HEP in River Klarälven. GH salmon and trout are used as broodstock and are stripped (i.e., artificially emptied of eggs and milt) to create the next generation of GH salmon and trout. Parameterization of fecundity (section ``Fecundity''.) and estimation of spawner lengths in the G-Rapids were done using these breeding data [29].

#### Electrofishing records

The open Swedish electrofishing register (SERS) hosts data from a range of environmental monitoring programs, including the County Administration's annual electrofishing surveys in the Gullspång River^1^. We used these data (which contains time and place of electrofishing, species, individual lengths, and estimated densities) for a range of applications in our parameterization.

##### Length distribution on electrofishing dates

**Fig. 3:**
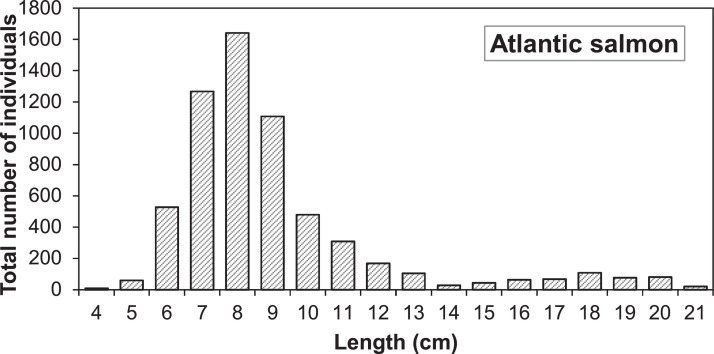
Length distribution of salmon caught after 1 September during electrofishing in the Gullspång Rapids (years 2004, 2005, 2008-2010, and 2013-2016), data from SERS [78].

**Fig. 4:**
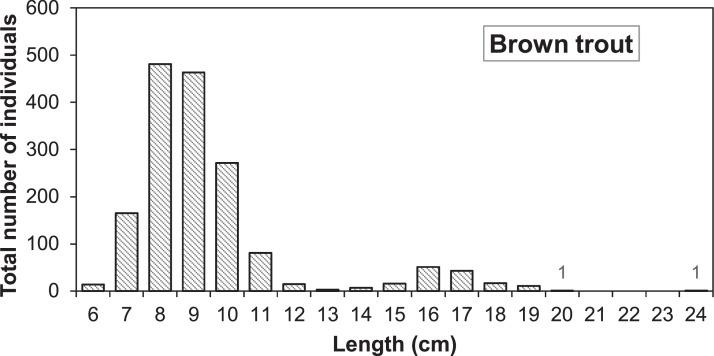
Length distribution of brown trout caught after 1 September during electrofishing in the Gullspång Rapids (years 2004, 2005, 2008-2010, and 2013-2016), data from SERS [78].

Electrofishing stations in the G-Rapids were established after the reach was restored, with the first survey taking place in 2004. The distribution of individual fish lengths in electrofishing after 1 September (i.e., after the main growing season) of 2004-2005, 2008-2010 and 2013-2016 show two distinct peaks, which we assume corresponds to the 0+ and 1+ cohorts (Fig. 3, Fig. 4). The most common size for 0+ fish is within the range 8.0-8.9 cm. We therefore assumed a length delimitation between 0+ and 1+ that were used for the parameterization. For salmon, we assumed a limit at 12 cm and for trout at 14 cm. In the future, the validity of this ageing based on length should be investigated more closely, as there is likely to be some overlap between age classes.

##### Density of juvenile fish

**Fig. 5:**
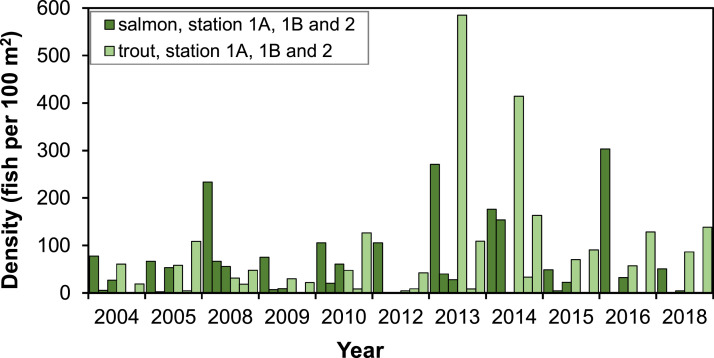
Density of juvenile salmon and trout in three regularly electrofished stations, data from SERS [78]. Years 2006, 2007, 2011 and 2017 are missing data and are thus not included on the x-axis.

**Fig. 6:**
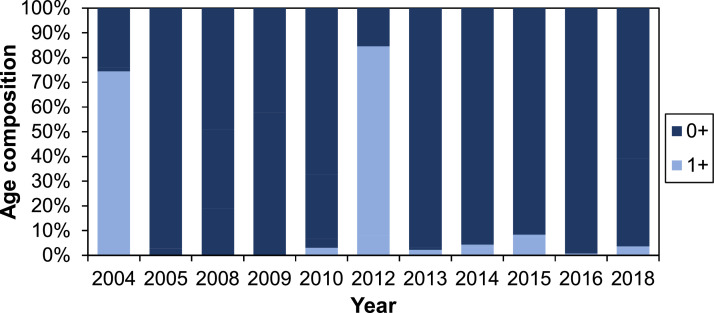
Density of 1+ relative to 0+ brown trout in the electrofishing data [78]. Years 2006, 2007, 2011 and 2017 are missing data and are thus not included on the x-axis.

**Fig. 7:**
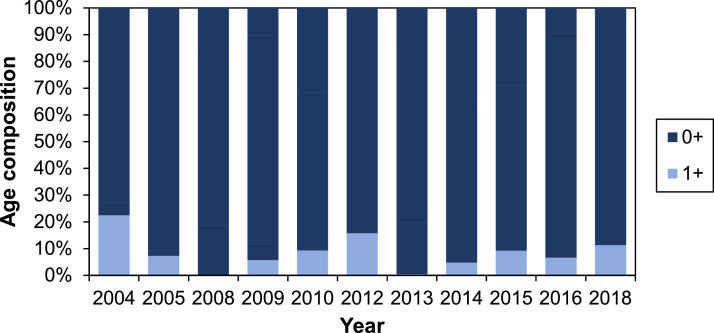
Density of 1+ relative to 0+ salmon in the electrofishing data [78]. Years 2006, 2007, 2011 and 2017 are missing data and are thus not included on the x-axis.

The density of juvenile salmon and trout in the G-Rapids varies both spatially and temporally, i.e., between electrofishing stations and years (Fig. 5). The overall trend seems to be an increasing density after 2010. The proportion of assumed 1+ fish caught during electrofishing is generally low (Fig. 6; Fig. 7), which may suggest that fish to some degree smoltify at 1+, or potentially even out-migrate during their first calendar year (i.e., as 0+). The high proportion of 1+ brown trout in 2012 can be explained by the large flood during the winter 2011/2012 that likely destroyed many of the trout redds (Fig. 6). Out-migration studies are however needed to understand more of the patterns of juvenile out-migration in the Gullspång River.

##### Presence of other species

Only 5 pike and 23 perch have been caught over ten years of electrofishing in the Gullspång Rapids [78]. These are likely to have come through the dam spill gates.

#### Redd surveys

Data from redd surveys was shared by Jukka Syrjänen. These surveys have taken place at the same time of the year and with approximately the same effort every year (in the beginning of December for one working week). During the surveys, redds are identified and verified by wading the river with aqua scopes and gently digging for live eggs in prospective redds for verification. Both Atlantic salmon and brown trout can sample spawning gravel by test digging on several locations without depositing eggs there (e.g., [28]). Samples of eggs or alevins have been taken from each redd for DNA (done by Luke/University of Helsinki) to establish annual salmon and trout breeding population size, as redds in the Gullspång River cannot reliably be assigned to species based only on physical characteristics (Jukka Syrjänen, pers. comm.). In addition to counting, mapping and taking specimen samples from redds, Syrjänen et al. [80] also registered the following physical characteristics of redds, which we used to calculate redd size (section ``Redd size'') and redd hydraulic suitability (section ``Depth and velocity''):

• Width and length of pot and tail, respectively
• Water depth over the longitudinal profile of the redd, three points in the pot and two points on the tail.
• Depth-averaged current velocity at the upstream and downstream edge of the pot, i.e., the same points as depth.
• Dominant substrate size just upstream of the pot, in the pot and in the tail, as well as the origin (natural or added during restoration) of the utilized spawning gravel.

#### Physical habitat surveys

During June 2018, we waded the Gullspång Rapids to map physical habitat features. We walked transects of the reaches, with 5-10 m spacing longitudinally, and registered point physical characteristics approximately at every 5 m of the transects. These were used for describing the cell's habitat (section ``Cell habitat file''). We registered:

• Dominant and secondary dominant substrate size.
• Availability of hiding cover and/or submerged vegetation.
• Depth-averaged water velocity (every 10 points).

### Observer variables and parameters

We ran the simulation over 10 years, starting on 20 September 2008 and ending on 20 September 2018. We chose these start and ending dates so that the model starts and ends immediately before adult spawners arrive in the reach, and because we have adequate biological survey data from 2008-2018. The selected period described ten hydrological years (typically defined from October to the end of September the following year).

### Habitat variables and parameters

The spatial entities in inSTREAM are reaches and cells. The G- Rapids model domain is a ca. 11700 m^2^ irregular polygon comprised of ca. 4500 equally sized hexagonal cells. The reach is curved, with a total length of ca. 270 m (measured as the midline of the wetted area). There are reach-specific parameters and input data as well as habitat cell input, with the cell being the smallest spatial resolution.

#### Reach-specific parameters

We have only one reach in this application, thus all reach parameters refer to the G-Rapids. Due to lack of site-specific information, we kept the suggested parameter values for prey energy density *habPreyEnergyDensity*, the downstream distance for regenerating drift depleted by foraging fish *habDriftRegenDist*, the number of hours that daytime extends from dawn and into dusk *habTwilightLength*, and the fraction by which water velocity is reduced in velocity shelters *habShelterSpeedFrac*. The shear parameters *habShearParamA & B* were roughly estimated, as we think redd scour rarely causes egg mortality in the G-Rapids. For other model applications where scour might be an issue, care should be taken to estimate these highly site-specific parameters. Due the G-Rapids being hydraulically complex, we assume that high flow does not limit spawning *habMaxSpawnFlow*, but rather that salmon and trout will attempt spawning for the whole range of discharges included (0.5-30 m^3^/s). The parameter for benthic prey production *habSearchProd* was calibrated (section ``Calibration of mean length of 0+ fish''), while the survival probability from aquatic predation (*habMortFishAqPredNightMin* and -*DayMin*) were estimated (section ``Fish survival from aquatic predation''). Table 3

**Table 3:** Reach-specific parameters and values for the G-Rapids.

Parameter	Value
habMortFishAqPredNightMin	0.99
habMortFishAqPredDayMin	0.98
habPreyEnergyDensity	4000 j/g
habDriftRegenDist	1000 cm
habShearParamA	0.001 s/m^3^
habShearParamB	0.9
habSearchProd	1.75E-06 g/h-cm^2^
habTwilightLength	0.5 h
habShelterSpeedFrac	0.5
habMaxSpawnFlow	30 m^3^/s

#### Reach temperature

In our model application, we could have used hourly time-series input of reach-specific temperature instead of daily. The temperature input file should contain one row per hour in the model hour. In our case, these 24-hourly values are all the daily average temperature. We do not have observational river temperature data per hour, or at all, for most of the period (Table 4). We therefore estimated daily river temperature based on an assumed relationship with air temperature. In Bjørnås et al. [9], we experimented with slightly deviating (± 1°C) temperatures from these estimates.

**Table 4:** Background data for modeling air to water temperatures 2008-2018.

Measured	Period	Source
Air temperature	1 Aug 2008- 30 Jun 2019	SMHI^2^
Water temperature	26 Nov 2015- 21 Sep 2016	Jukka Syrjänen and Johnny Norrgård, pers. comm.
	17 Nov 2016- 30 Dec 2016	
	25 Feb 2019- 5 Jun 2019	Own data

The air temperature was measured at Åtorp metrological station ca. 20 km northeast of the Gullspång Rapids^2^. For estimation of water temperature, we used a simplified version of the model by Cluis [12]. This model has been reproduced for medium to large temperate rivers by for instance Caissie et al. [10] and Ahmadi-Nedushan et al. [3]. Our simplified model was however created by:

1. Fitting all available data on air temperature and water temperature over the years (Table 4) to a sinusoidal function describing the coarse yearly fluctuations in temperature.
2. Fitting a linear model of water temperature residuals from air temperature residuals.
3. Combining 1) and 2) to a full model.
4. Changing estimated negative water temperatures to zero.
5. Adding 0.5 to all values to avoid low temperature mortality on eggs.

#### Reach turbidity

Turbidity is an optical property of water that can be measured with a range of different methods and in different units. Other inSTREAM simulations have demonstrated the importance of turbidity as an environmental parameter for visually guided foragers like trout [39]. Unfortunately, water quality monitoring programs in the Gullspång River do not include regular turbidity measurements. Instead, we investigated a turbidity data series from a measuring point some hundred meters upstream of the dam from the environmental database Vattenwebb^3^. These measurements indicated a very low turbidity year-round (0.5-3.5 Formazin Nephelometric Units (FNU)). In inSTREAM, turbidity effects are ignored for turbidity values < 5 Nephelometric Turbidity Units (NTU), which is similar to FNU. We therefore ignored turbidity in our application.

#### Reach discharge

**Fig. 8:**
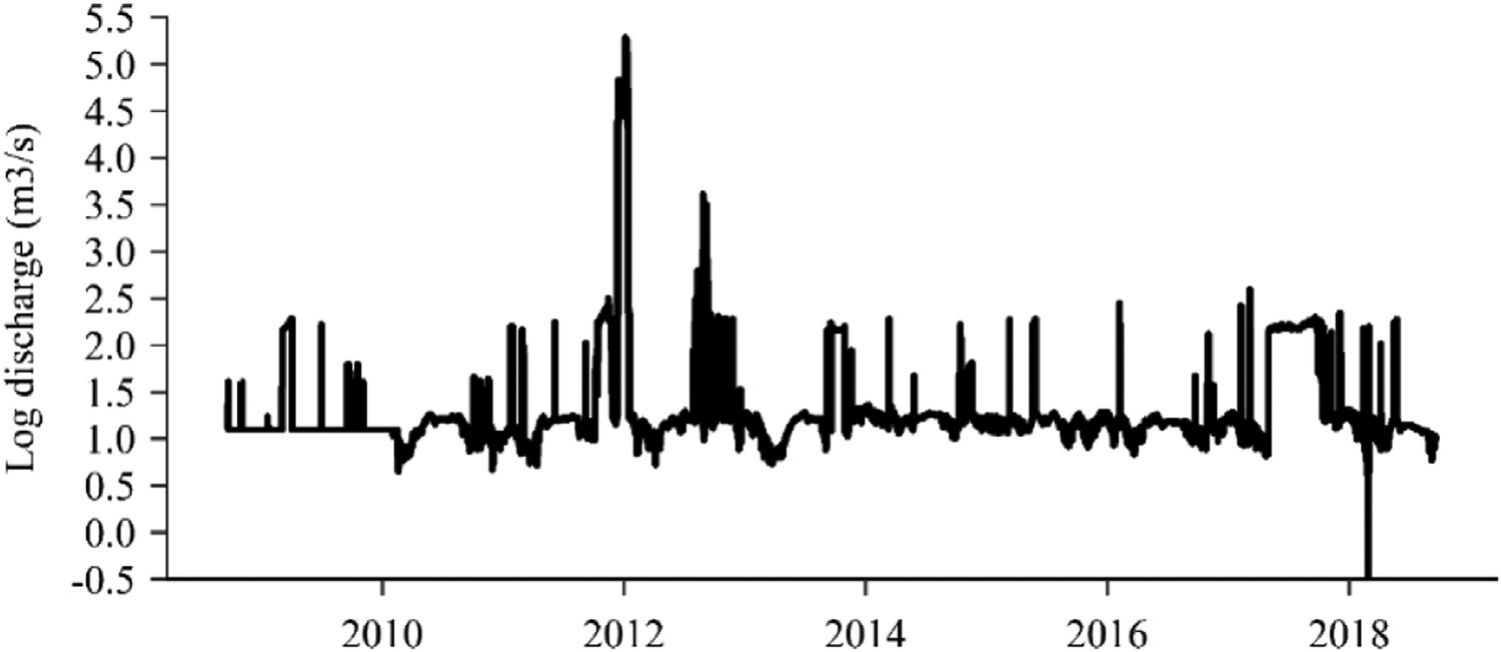
Log-adjusted flow through the spill gates to the Gullspång Rapids during the simulation period 2008-2018. Note the high flow peak in 2011/2012.

As explained in section 1.5, discharge is the main environmental factor we vary in this application. Discharge to the G-Rapids is not constantly at the minimum flow, but rather varies slightly depending on the water level in the reservoir and on the operational status of the power plant. The dam gates have a fixed opening subsurface, and by running the turbine intake there will be a drop in surface elevation between Lake Skagern and the dam (Claes Kjörk, pers.comm.). Fortum provided hourly discharge through to the G- Rapids for the simulation period, from which all input files for the null scenario and minimum flow scenarios (section ``What are the effects of increasing the minimum flow?'') were based. The data was recorded using summer and winter time zones, i.e. with one extra hour at the end of each October and one missing hour at the end of each March. To make the time series compatible with inSTREAM, we removed the superfluous hour in October and inserted the missing hour, using the same flow value as the hour before, in March. Because the hydraulic MIKE model became unstable and unsolvable at flows above 30 m^3^/s, we disregarded the extreme flow event of 2011 (Fig. 8). During 6441 h, the flow exceeded 30 m^3^/s, and we replaced these values with 30 m^3^/s. This became the *Flow0* default flow input. The discharge to the G-Rapids has occasionally dropped below 3 m^3^/s during the period 2008-2018. We explored if these low flows had any effect on the population in the *Flow3* scenario. To make this scenario's flow input file, we copied the flow input from *Flow0* and replaced all discharges < 3 m^3^/s with 3 m^3^/s. The same was done with the 6 m^3^/s minimum flow scenario *Flow6,* and the 9 m^3^/s minimum flow scenario *Flow9.*

The natural flow input (section ``What are the effects of a more natural flow regime?'') were prepared by downloading S-HYPE model data for the Gullspång River [79]. From the column of total natural discharge (mean daily), we extracted the time series for our model period and prepared input files for SHYPE1, SHYPE2 and SHYPE3 with 10%, 20% and 33% of the daily discharge values, respectively.

#### Arrival of spawners to the G-Rapids

As explained in section 2.7., arrival of adult salmon and trout to the spawning grounds is specified as an arrival input file. We varied the number of arriving spawners per year of the two species in adult return experiments. The null scenario was however 20 spawners per year and species. We created arrival files with 20 and 40 returning adults per year for each species, while keeping the arrival dates and distribution the same.

We had some information on the timing of arrival of spawners in the G-Rapids based on our own and others’ observations throughout the years. In addition, we could deduce spawning time deduced from egg development stage during the redd surveys in December [80]. We assumed that adults arrive shortly before they spawn, some even after the first individuals of their species have started spawning. Therefore, we decided to spread out salmon and trout arrival evenly over the course of 20 days: ten days before and ten days after the *fishSpawnStartDate*. Therefore, trout were set to arrive September 25 - October 14 and salmon on October 22 - November 10 (Table 5).

**Table 5:** Calendar to illustrate spawning events. “BT” are trout and “AS” are salmon arrival dates. Yellow is spawning period for trout exclusively, green overlapping spawning days, and blue exclusively salmon spawning days.

**Fig. 9:**
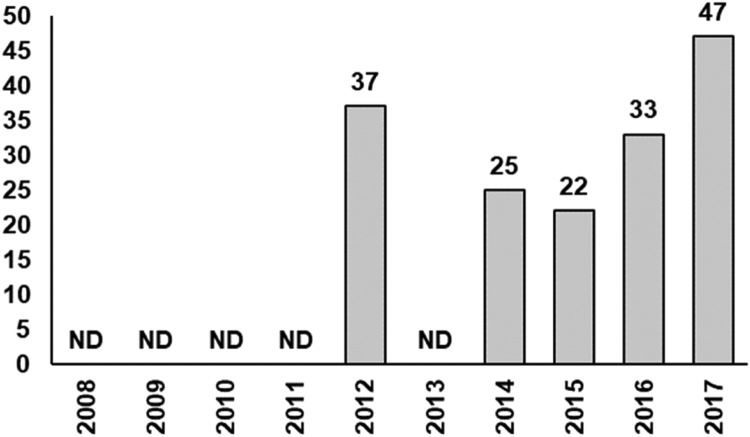
Total number of redds found in the Gullspång Rapids, by Jukka Syrjänen and others, during the model period 20 September 2008- 20 September 2018. Note that in half of the years, there were no redd surveys (ND= no data) in the G-Rapids.

The number of redds gives some indication of the total numbers of females per year (Fig. 9), at least after 2014 when the annual surveys were done with comparable methods (section ``Redd surveys''). We did not use redd count data directly because many redds could not successfully be identified to species due to low DNA content of sampled eggs (Jukka Syrjänen and Johnny Norrgård, pers. comm.). For Atlantic salmon and brown trout, one observed redd does not always correspond to one spawning female as it can occur that a female places eggs in several redds [7]. Therefore, we based the null scenario on the number of genetically effective spawning pairs per year and species described by Palm & Dannewitz [57]. Assuming the exact same number of returning adults per year is an unrealistic model simplification that enables comparison of 0+ cohort success between years, as the preconditions in terms of spawners are the same.

**Fig. 10:**
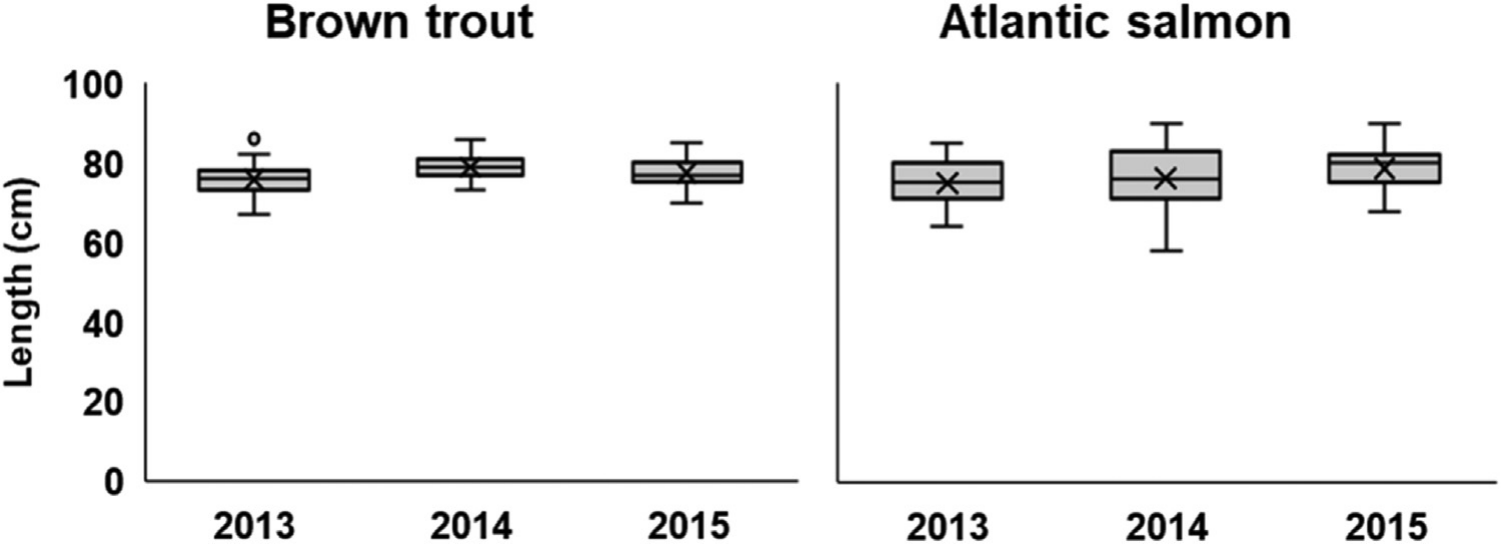
Length distribution of hatchery-raised female brown trout and Atlantic salmon of Gullspång origin caught in 2013, 2014 and 2015 in the spawner trap in Forshaga, River Klarälven.

We had no data on the numbers and length distribution of salmon and trout spawners. Instead, we assumed that the size distribution at spawning migration of hatchery raised Gullspång salmon and trout trapped in River Klarälven (section ``Ex situ populations in River Klarälven'') are representative for their corresponding wild populations in the Gullspång River. We used data on lengths of salmon and trout when stripped (i.e., emptied of eggs and milk). The length distribution in the available dataset from 2013-2015 differed little between years (Fig. 10), so we pooled the data. Overall, the mean length (± SD) of female salmon (N = 185) and trout (N = 179) was 76.79 (± 5.90) and 77.38 cm (± 3.60) cm, respectively. These values were used for all years in the arrival input files. Note that this length distribution represents first-time spawners, as these individuals are caught on their first spawning migration.

We assumed that adult arrival happens evenly spread out during the 20 assigned days of the arrival period (Table 5). All arriving fish were assigned the age of four calendar years because as inSTREAM 6.1 (being developed for resident fish) require fish to be at least 4 years old to spawn.

#### Drift food concentration

InSTREAM 6.1 uses hourly input files of drift concentration, measured in g/cm^3^. As we had no time-series data on drift concentrations, we used a constant concentration and treated it as a parameter value. The drift food concentration was calibrated, together with search food production (section ``Reach specific parameters''.) and terrestrial predation survival (section ``Minimum terrestrial predation survival day and night''), to mean lengths of 0+ fish in section ``Calibration of mean length of 0+ fish''.

#### Cell geometry file

InSTREAM requires three cell input text files: a geometry file, a habitat file and a hydraulics lookup file. The geometry file contains the corner coordinates and ID number of each cell. As the cells are the smallest unit in inSTREAM, it is important how they are represented. Cells should be small enough to capture major hydraulic variation but large enough to fit salmon and trout redds, as well as large enough to provide enough resources for a superindividual object. “What is the best scale?” is a recurring question within the interdisciplinary field of ecohydraulics (e.g., [49,51]). We highlight that inSTREAM is predominantly an ecological model, where ecologically relevant hydraulic complexity is represented in built-in factors such as any cell containing a proportion velocity shelter (section ``Velocity shelter availability'').

**Fig. 11:**
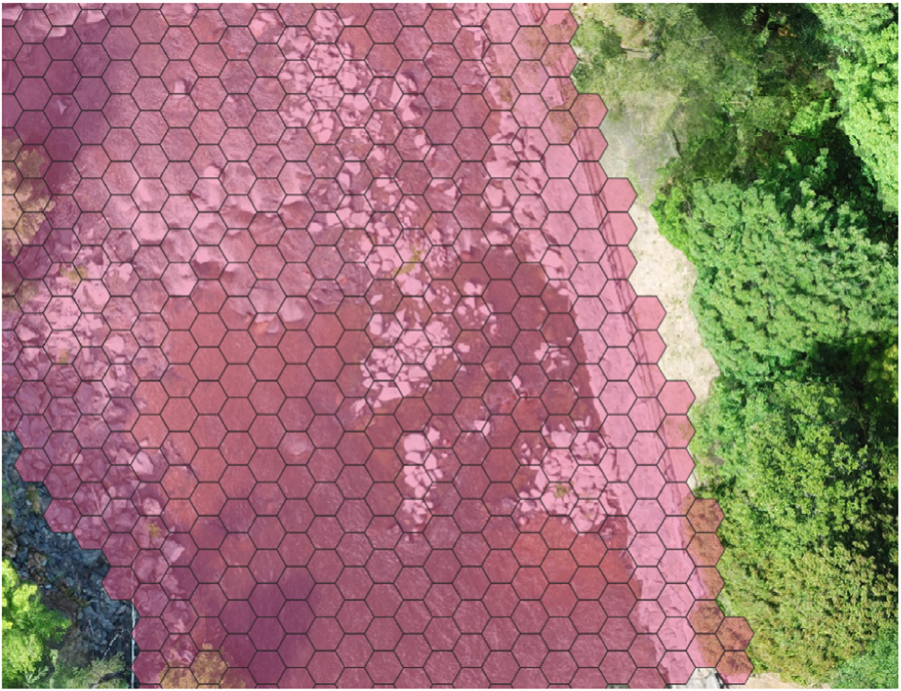
The uppermost G-Rapids: zoomed in on a section of the reach showing the hexagonal inSTREAM cells (pink) overlaid an orthomosaic image.

We created a hexagonal grid layer covering the model area in QGIS 3.4 [63] using the MMQGIS plugin, setting the width in the east-west direction to 1.5 meters (Fig. 11). The result was 4503 cells each with an area of 2.598 m^2^. The model area polygon was the same used to delimit the simulated area in the hydraulic model. This grid layer was used in all consecutive steps. To create the cell geometry file on the correct format for inSTREAM, we used the QGIS 3.4 plugin “Geometry editor” (Peter Dudley; www.github.com/pndphd/geometry_generator). This plugin creates a text file containing cell number and corner coordinates of respective cells.

#### Cell habitat file

The cell habitat file consists of the variables cell ID, fraction velocity shelter, distance to hiding cover, fraction spawning gravel, and fraction hiding cover as well as reach end id. We drew polygons of central features of the fish habitat in QGIS 3.4 using surveyed habitat points (section ``Physical habitat surveys'') as guidance. We also created the hexagonal inSTREAM cells in QGIS and merged them with the habitat layers.

##### Cell ID

The cell ID is a unique integer attribute of each hexagon that is used to link cell habitat, geometry, and hydraulics files. The cell ID can also be used to trace back output such as redd placement to their explicit location in the reach. We used the feature ID numbers automatically generated for the hexagons as cell ID numbers.

##### Velocity shelter availability

Velocity shelters in InSTREAM (*cellFracShelter*) are areas behind instream structures, where the water velocities locally are lower so that fish can save energy while drift-feeding. Velocity shelters can be on a finer scale than the cells and should not be considered for the cell's averaged hydraulic properties. The amount of velocity shelter area in each cell is included in the cell habitat file as a fraction between zero and one of the cell's total area, and this fraction stays constant over the whole simulation period.

The distribution of velocity shelters was determined and defined in QGIS by visually inspecting an UAV orthomosaic layer and drawing polygons in a designated velocity shelter layer. We treated areas in the downstream side of boulders, wood and concrete structures as velocity shelters. In total, we defined 186 m^2^ of the model area as velocity shelter. The velocity shelter layer and the cell definition layer was combined using an intersection analysis in QGIS in order to calculate the fraction of velocity shelter in each cell.

##### Fraction hiding cover

Hiding cover in inSTREAM (*cellFracHidingCover*) is the proportion of a cell's area that provides hiding possibilities for a juvenile salmonid. Hiding cover can be e.g. aquatic vegetation, instream wood and large boulders. To represent hiding cover we drew polygons based on the UAV ortomosaic photos, treating areas with large boulders with interstitial space, overhanging trees and other vegetation as hiding cover. Aquatic vegetation is almost non-existent in the reach. We defined in total 3006 m^2^ of the model area as hiding cover. The calculation of fraction hiding cover in each cell followed the same procedure as for fraction velocity shelter and fraction spawning gravel.

##### Distance to hiding cover

A fish engaged in feeding is exposed and has a higher risk of being discovered by predators. Having somewhere to hide nearby can increase survival from pursuit predators. We defined the distance to hiding cover for a cell (*cellDistToHide*) as the closest distance between the centroid of the cell and the nearest hiding cover polygon. We first created a new layer of cell centroids using the QGIS Vector tool ‘Centroids’. Then we used the plugin NNJoin to find the nearest neighbor distance between the centroids (as ‘input’) and hiding cover (as ‘join’). The attribute table of the resulting layer was exported and merged with the rest of the habitat input data using the Excel Power Query tool.

##### Fraction spawning gravel

Availability of gravel within the right size range is essential for successful reproduction for both Atlantic salmon and brown trout. “Gravel” in this paper represents all sediment size classes used by spawning Gullspång River salmon and trout (not necessarily “gravel” in e.g. the Wentworth scale). Addition of spawning gravel is a common restoration action in rivers where the natural sediment transport regime has been disturbed [6]. The G-Rapids are located immediately below a large dam and thus spawning gravel is not recruited naturally.

The cell input attribute spawning gravel fraction (*cellFracSpawn*) was calculated by drawing polygons over the spawning gravel beds, as they were located after addition in the fall of 2018, which were intersected with the habitat cell layer. We drew the spawning gravel beds based on visual inspection of ortophotos, consultation with managers and field validation during redd counts in December 2018. The area of polygons representing spawning gravel amounted to a total of 773 m^2^. This area could theoretically fit 37 redds without overlap with our defined *reddSize* (section ``Redd size'').

##### Reach end ID

Cells that constitute the upstream and downstream borders of the model area must be assigned as such. The habitat file therefore has a column for whether a cell is located inside (I), at the upstream (U) or at the downstream (D) end of the reach. The upstream and downstream cells should be flooded during normal flow, while the rest of the cells are defined as being inside. We assigned cells to these categories using the orthomosaic as guidance. The reach end ID information was merged with the other habitat properties using cell ID as the unique identifier.

#### Cell hydraulics file

The cell hydraulics file contains cell-by-cell average values of depth and velocity for a range of flows. Cells’ hydraulic properties were averaged from the hydraulic model solutions in MIKE 21 Flexible Mesh Flow Model [17]. To develop the hydraulic model of the G-Rapids, we made an elevation model of the riverbed and riparian zone from bathymetry points surveyed in 2018, and Lidar data from the Swedish National Elevation model [50]. The resulting elevation model was used for setting node elevations on a self-defined computational mesh in the Mike Mesh Generator, a mesh that was used for hydraulic simulations in MIKE 21, for twenty discharges ranging from 0.5 m^3^/s to 30 m^3^/s.

MIKE 21 FM numerically solves two-dimensional shallow water equations using a cell-based finite volume method. The mesh of the hydraulic model consisted of near 40 000 elements to capture fine scale hydraulic properties on a scale much smaller than inSTREAM cells. This was necessary in order to get stable hydraulic solutions in MIKE. We performed hydrodynamic simulations over 7200 time steps of 1 s with the upstream boundary conditions of the following discharges: 0.5, 1, 1.5, 2, 2.5, 3, 3.5, 4, 5, 6, 7, 8, 9, 10, 11, 12, 15, 20, 25 and 30 m^3^/s. The downstream boundary condition was set to an assumed constant water surface elevation of 49 m a.s.l., with the downstream boundary being far downstream the reach modelled in inSTREAM (to avoid edge effects). For each hydrodynamic solution, we transformed the native MIKE-format to shape files of either depth or velocity at the very last time step.

The shapefiles were imported into QGIS, rasterized and renamed. The rasterize operation was done with the GDAL algorithm, using the model area as extent, raster pixel size 0.1 × 0.1 m and depth/current velocity as the burn-in value of the single band. When all flow solutions (20 discharges × 2 hydraulic properties) had been rasterized and renamed we created the cell hydraulics file. The depth and velocity in the raster pixels were averaged within the overlaid hexagonal cells and written to a tex tfile on the format for inSTREAM. We used the QGIS plugin ‘Hydro Generator’ by Peter Dudley.

### Fish variables and parameters

Both species of fish and redds are described by the same state variables and parameters, but some parameter values vary between species. All fish-related variables and parameters are presented here in the order of the submodel/method to which they are related.

#### Decide whether to spawn

Salmon and trout arrive to the G-Rapids during the fall to spawn, after having grown to maturity in Lake Vänern [75]. Trout spawners are the first to arrive to the reach, while salmon spawners arrive some weeks later. Based on openly available electrofishing records [78], we assume that there are very few resident trout in the area. Some smaller individuals (presumably precocious males) have been observed during spawning (own observations), but for inSTREAM, the size of male spawners is irrelevant. To our knowledge, no small redds (indicating smaller, resident females) have been found in the G-Rapids, so we assume that all salmon and trout females follow the lake migration life-history strategy.

Female Atlantic salmon and large brown trout is known to spread egg mortality risk by making several redds, often with multiple males siring their offspring [7,^23^,28], and studies of egg/alevin DNA from redds suggest that this occurs to some degree in the G-Rapids. One redd produced per female is however a necessary simplification of inSTREAM 6.1, as the mechanism behind why a female choose to deposit eggs in one or in more redds to our knowledge is unknown. In nature, female Atlantic salmon choose a spot to make a redd and defend this spot from being used by other females whilst sampling and excavating gravel and depositing and covering eggs. This process that can last from an hour to two days [28]. Females’ relatively short-term defense of spawning locations is not included in inSTREAM, as spawning is coded as an instantaneous event (Schedule 2.2.2. step 6A).

The redd surveys suggest that some degree of superimposition occurs in the G-Rapids with different egg/alevin developmental stages sometimes occurring within the same redd (Jukka Syrjänen, pers. comm.). Superimposition can be a natural consequence of the long, combined spawning period that stretches over several months, limited areas with spawning gravel and no post-spawning redd guarding.

##### Spawning criteria

In addition to being limited by external conditions, spawning is constrained by the internal state of the fish. To spawn, a fish needs to have reached a certain age (*fishSpawnAge*), length (*fishSpawnMin-Length)* and condition factor (*fishSpawnMinCond*). These parameters are associated with the resident trout version of the model. As we model a discontinuous life cycle, we already control the length, age, and condition of adult spawners (section ``Arrival of spawners to the G Rapids''). We therefore set the spawning criteria parameters so that adult fish can spawn while juvenile fish cannot (Table 6).

**Table 6:** Parameters for minimum age, condition, and length of spawners

Parameter	Value (both species)
fishSpawnMinAge	4 years
fishSpawnMinCond	0.5
fishSpawnMinLength	25 cm

##### Spawning season date window

Model fish can only spawn within a period defined by the parameters *fishSpawnStartDate* and *fishSpawnEndDate* (Table 7). Ros [75] reported that brown trout arrive at the spawning sites in the Gullspång River in October, and that spawning occurs from mid-October to early November, while salmon migrate to spawning sites from early October to mid-November, and that they spawn from November to early December. The County Administrative Board have observed first spawning between 8 October and 17 October ([30] Table 9), and we have observed spawning activity as early as 5 October. We therefore allowed the date window to be wide. Additional clues as to when trout and salmon are ready to spawn can be deduced from the hatchery stripping data (section ``Ex situ populations in River Klarälven''). In 2013-2015, GH trout were stripped between 13 October and 6 November, while GH salmon were stripped between 3 November and 11 December.

**Table 7:** Parameters for salmon and trout spawning criteria.

Parameter	Value trout	Value salmon
fishSpawnStartDate	10/05 (5 Oct)	11/1 (1 Nov)
fishSpawnEndDate	11/10 (10 Nov)	12/05 (5 Dec)
fishSpawnMaxTemp	10	10
fishSpawnMinTemp	5	5
fishSpawnMaxFlowChange	1	1
fishSpawnProb	0.5	0.5

##### Temperature criteria for spawning

The temperature range in which the fish will spawn is given by the parameters *fishSpawnMaxTemp* and *fishSpawnMinTemp.* In inSTREAM, temperature extremes are thought to be a cue that delay spawning to avoid temperature-induced egg mortality. Jonsson & Jonsson [44] noted that spawning activity of both Atlantic salmon and brown trout ceases when temperature drops below 5°C. Likewise, we assume that fish avoid spawning when the water temperature is above the upper limit of optimal temperature for egg development. For brown trout this temperature is ca. 10°C [56], and we assume the same temperature is valid for salmon (Table 7).

##### Flow changes

Fish might delay spawning when flow fluctuates drastically between days. If the fractional flow change exceeds the parameter *fishSpawnMaxFlowChange*, a unitless fraction, no fish will spawn on that day. This parameter is not relevant at our study site, which experiences an almost constant flow and less drastic flow changes due to the dam. Thus, we set this parameter to the maximum flow value (Table 7).

##### The probability of spawning on a given day

Whether or not a female spawns when all criteria for spawning (this section and section 3.4.1.) are fulfilled, is determined stochastically. This imposes some variation in spawning date. Early spawning can lead to a competitive advantage for the offspring as they will emerge from the gravel and start feeding early [21]. However, early spawning can also be a disadvantage if river discharge fluctuates strongly in the early part of the season (during fall floods), leaving eggs vulnerable to mortality from redd scour and desiccation. Redds that are made early may also risk superimposition by later spawners. The parameter *fishSpawnProb* is the probability that a fish will spawn on a day when conditions otherwise are suitable. The parameter value was set high (Table 7) to make adults spawn shortly after they arrived (section ``Arrival of spawners to the G Rapids'').

#### Select spawning cell and move here

Depth, velocity and substrate are the most important microhabitat features of the spawning habitat [28]. Substrate in the form of spawning gravel is included as habitat cell input data (section ``Fraction spawning gravel''), while depth and velocity suitability are fish parameters. Due to difficulties in assigning species to individual redds, we use the same redd site selection parameters for salmon and trout. In the model, the quality of a potential spawning cell (*spawnQuality)* is the product of suitability index values for depth and velocity, the area of gravel in the cell, and an uncertainty:spawnQuality=spawnDepthSuit×spawnVelocitySuit×spawnGravelArea×U where U is one plus the product of a random decimal number between zero and one and the parameter *fishSpawnCellUncert:* U=1+Rand(0,1)*fishSpawnCellUncert. The increase in spawning quality is thus in the range of zero and the value of *fishSpawnCellUncert*. We used a low value for *fishSpawnCellUncert,* 0.15. This new parameter was also subject to a sensitivity analysis (section ``Spawning cell selection uncertainty fishSpawnCellUncert'').

##### Depth and velocity

A practical advantage of the stable flow at the Gullspång Rapids is that the hydraulic conditions during redd surveys (section ``Redd surveys'') are similar to those when the spawners were assessing the reach and selecting a location for spawning. We therefore used measurements of velocity and depth recorded during the redd surveys in 2012, 2015 and 2018 [80], which were the years with the most stable flows in the fall. Density function curves were created using R [64] and standardized to an index between zero and one.

**Fig. 12:**
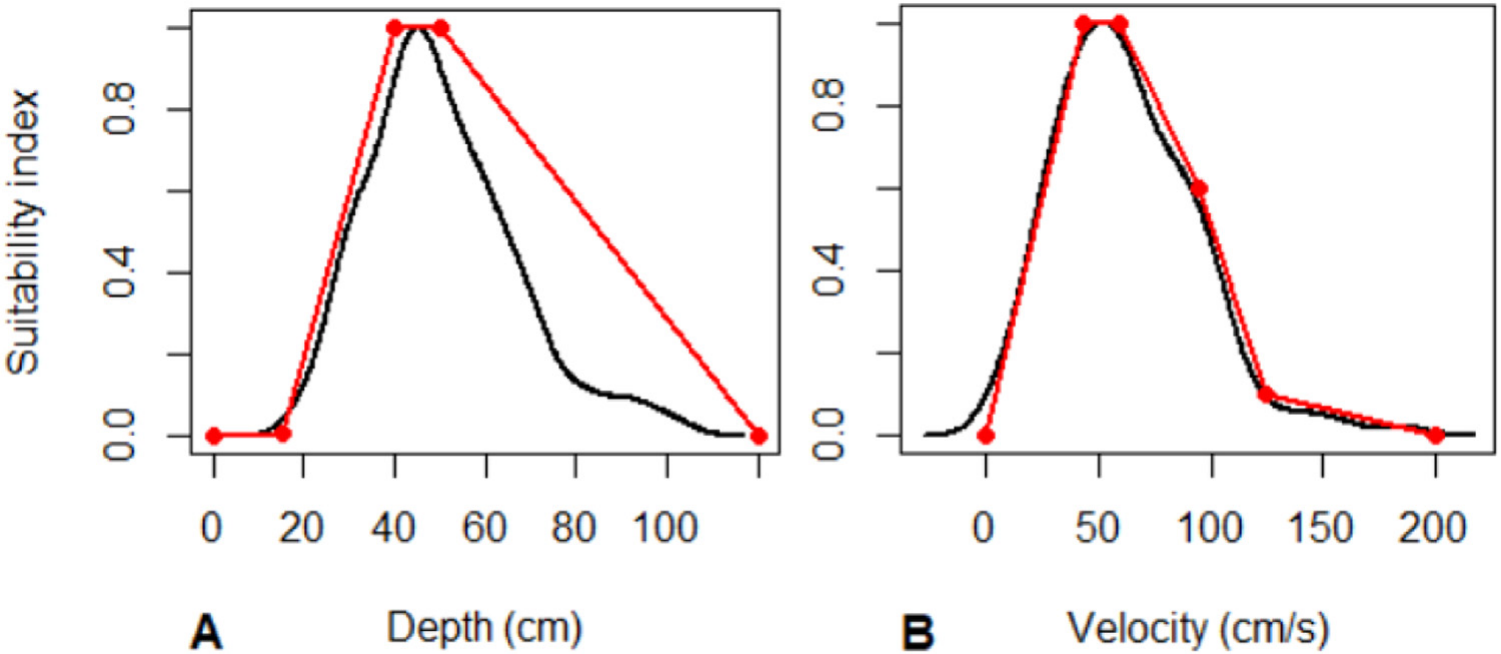
Redd suitability curves (red) for both salmon and trout redds in the Gullspång Rapids. A) Depth suitability, based on observed depth at the upstream edge of redds (black). B) Velocity suitability, based on observed velocities at 0.6 of depth (measured from the surface) at the upstream edge of redds (black). Data from Syrjänen et al. [80].

The depth at the most upstream edge of 512 redds was plotted in a density plot (Fig. 12A), where density values represented a relative suitability index. We added five points, between which we assumed linear relationships between depth and suitability, to reproduce the curve. The coordinates of these five points were used as the depth suitability parameters (Fig. 12A; Table 8). Similarly, the velocity at 0.6 of the depth (measured from the surface) of the same 512 redds was plotted in a density plot (Fig. 12B). The 0.6 depth is considered the average water velocity over the depth profile ([2], p.107). We reproduced the curve (similarly as for depth) using six points. These points were used as parameter values (Fig. 12B; Table 8)).

**Table 8:** Parameters for depth and velocity spawning suitability.

Depth	Value (cm)	D suitability	Value
fishSpawnDSuitD1	0	fishSpawnDSuitS1	0
fishSpawnDSuitD2	15	fishSpawnDSuitS2	0.01
fishSpawnDSuitD3	40	fishSpawnDSuitS3	1
fishSpawnDSuitD4	50	fishSpawnDSuitS4	1
fishSpawnDSuitD5	120	fishSpawnDSuitS5	0
**Velocity**	**Value (cm/s)**	**V suitability**	**Value**
fishSpawnVSuitV1	0	fishSpawnVSuitS1	0
fishSpawnVSuitV2	43	fishSpawnVSuitS2	1
fishSpawnVSuitV3	60	fishSpawnVSuitS3	1
fishSpawnVSuitV4	95	fishSpawnVSuitS4	0.6
fishSpawnVSuitV5	125	fishSpawnVSuitS5	0.1
fishSpawnVSuitV6	200	fishSpawnVSuitS6	0.1

As the suitability curves are based on observations of actual redd placements, they also reflect the microhabitat availability in the reach at the times when redds were made and the size and swimming capacity of the spawners those years. The suitability curves are therefore site-specific. The curves are more uncertain for interpolations to a higher minimum flow that would alter the hydraulic landscape. Furthermore, microhabitat features such as velocity shelters might allow for actual spawning in cells where the simulated water velocity is unsuitable, but our model does not account for this effect of velocity shelter.

#### Create a redd; set number of eggs

Female salmon and trout deposit their eggs into an excavated gravel pit, with a male that stands beside and releases milt. The female then covers the egg pocket the same way the gravel pit was dug, i.e., with rapid movements of the tail fin [28]. The resulting structures (redds), provide a protected microhabitat for the eggs and alevins in the hyporrheic zone while they develop, hatch and consume their yolk sac. The physical structure of redds locally alter the three-dimensional water velocity field and oxygen exchange pattern [82].

##### Redd size

**Fig. 13:**
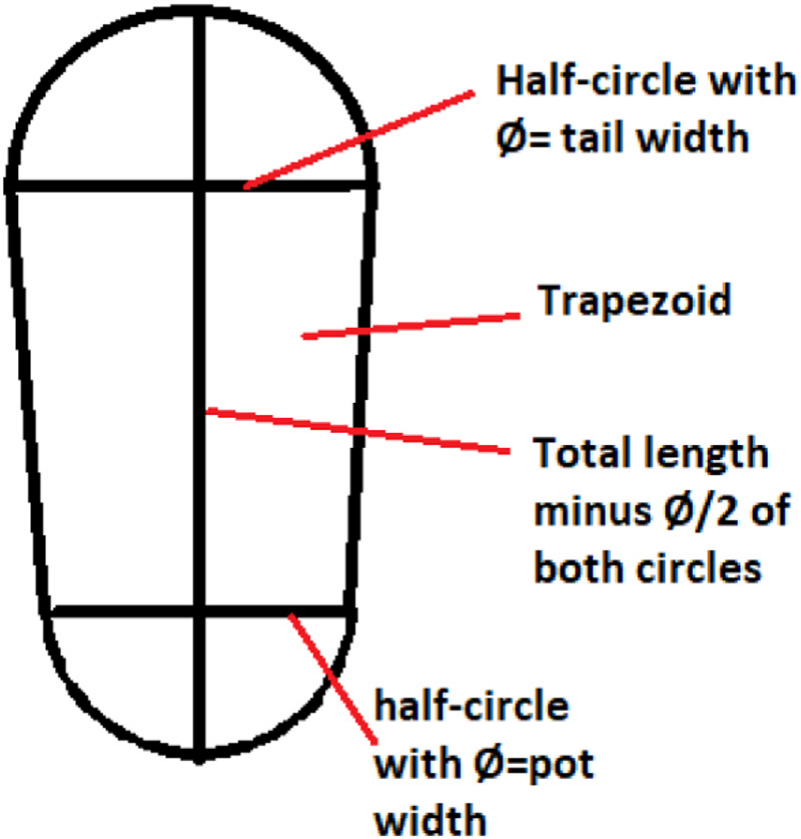
Simplified redd geometry in the form of to two half-cirles and a trapezoid.

The inSTREAM parameter *reddSize* is defined as the area that a spawner disturbs when digging a redd. This area increases with the size of the females (e.g., [15]). We used data on redd sizes from the whole river in 2018 (N=183). The ongoing redd survey program consists of locating redds right after the last fish are assumed to have spawned, i.e., in early December each year. The length and width of pot and tail and other physical and hydraulic characteristics (section ``Redd surveys'') are measured for every redd [80]. Redds made by similarly sized Atlantic salmon and brown trout are virtually indistinguishable in terms of location and size (Syrjänen, pers.comm.), so we used the same size parameter estimate for both species. We estimated the two-dimensional horizontal area of redds as the sum of two half-circles and a trapezoid (Fig. 13). The mean and median redd sizes were 24896 cm^2^ and 20790 cm^2^, respectively. As the size distribution was skewed towards smaller redds, we used the median size as the parameter value for *reddSize* (Table 9).

**Table 9:** Parameter values related to creation of redds and deposition of eggs.

Parameter	Value trout	Value salmon
reddSize	20790 cm^2^	20790 cm^2^
fishFecundParamA	2.267	5210
fishFecundParamB	1.746	0
fishSpawnEggViability	0.9	0.9

##### Fecundity

The fecundity parameters relate a female's length to the number of eggs deposited: $\mathrm{numb}\mathrm{er}\;\mathrm{of}\;\mathrm{eggs}\;\mathrm{prod}\mathrm{uced}=\mathrm{fish}\mathrm{Fecu}\mathrm{ndPa}\mathrm{ramA}\times \mathrm{fish}\mathrm{Leng}h^{\mathrm{fish}\mathrm{Fecu}\mathrm{ndPa}\mathrm{ramB}}$ . We fit the two fecundity parameters (A and B), using data from stripping of GH salmon and trout (section ``Ex situ populations in River Klarälven''). The data contained information on date, size of female parents and of male parents per tray of mixed egg and milt (2-3 of each sex). In other words, the data on estimated number of eggs was not logged per individual, but per mix of 2-3 females. Egg size was estimated by Fortum Standardfiske by counting the number of eggs that could fit on line in a 250 mm long cylinder. Total number of eggs was estimated from egg size and total volume and rounded to the nearest 50 eggs (Johnny Norrgård, pers. comm.). To minimize potential error from aggregating eggs of several individuals, we filtered the data by discarding records where the length difference of the females in a pair was ≥3 cm and where eggs of three females had been pooled together. We also discarded records where the egg diameter was outside the average ± SD (trout: 6.10 ± 0.25 mm; salmon: 6.20 ± 0.26 mm)

**Fig. 14:**
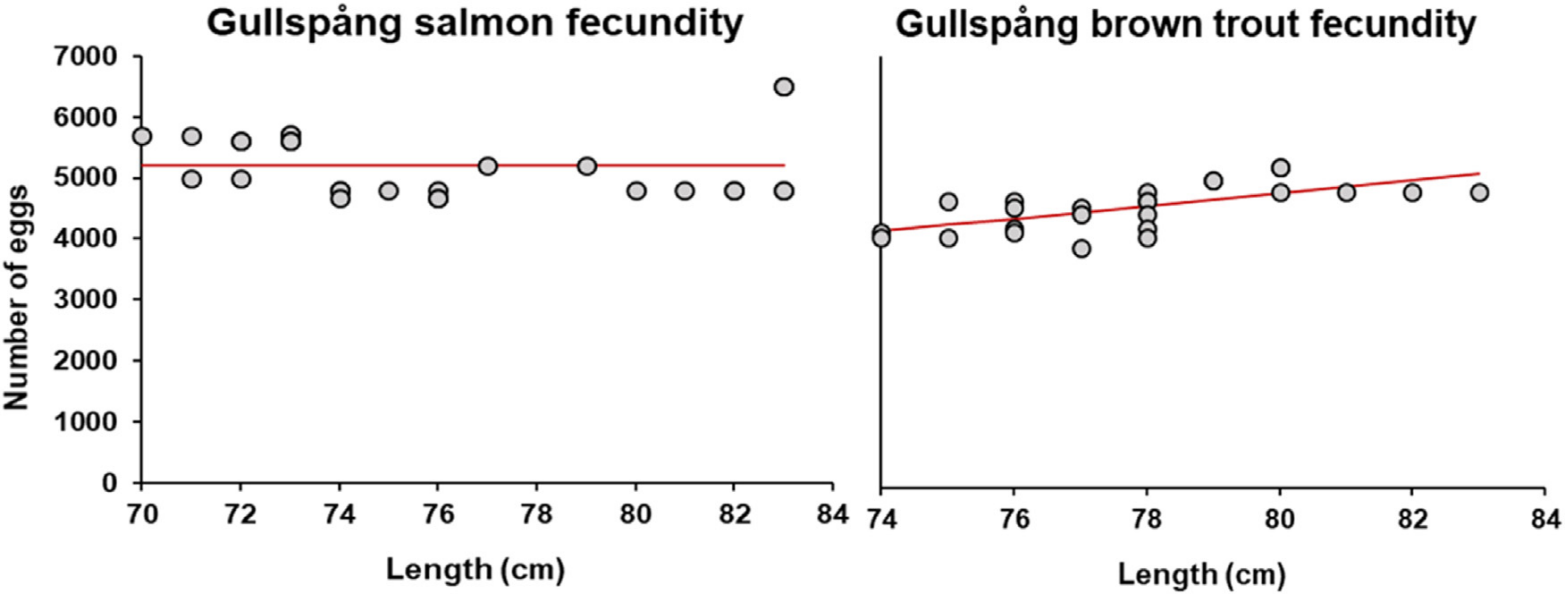
Observed relationship between number of eggs and fish length (grey points) and fitted model with the two fecundity parameters (red line). For salmon, the parameter combination that gave the best fit to the data was a constant.

We fitted the parameters using the GRG Nonlinear algorithm to minimize the sum of squares between observed and predicted in Microsoft Excel Solver. We sorted the records and discarded the 25% where fecundity was overestimated to take into account that stripping does not always empty the female of all eggs. Lastly, we repeated the parameter fitting on the remaining records of 25 brown trout and 27 salmon (Fig. 14; Table 9).

Fecundity is likely to be slightly site-specific, partly because of the substantial intraspecific variation in egg size in salmonids. Larger females often have disproportionately fewer, but larger eggs than smaller females, i.e., there is a positive relationship between investment per offspring and female size [74]. This was also detected in the stripping data. Another general pattern is that southern populations produce proportionally larger and fewer eggs than northern populations (Bror Johnsson, pers. comm.). Our fitted parameters for brown trout gave much lower fecundity estimates than you would get following for instance the formula of van Winkle et al. [84] –which is natural because their parameters were developed for smaller, resident trout. Nevertheless, if local data is lacking, it might suffice to use standard fecundity parameter values as they usually have a small effect on the overall simulation results.

##### Egg viability

The parameter *fishSpawnEggViability* describes the proportion of a female's eggs that are successfully fertilized and placed in a redd. We do not have direct data on this proportion from the Gullspång River, but redd survey observations (where eggs are partly excavated) suggest a high proportion of viable eggs shortly after fertilization. We therefore estimate that 90% of the eggs are viable (Table 9).

#### Incur weight loss

After spawning, both females and males incur a weight loss represented by the parameter *fishSpawnWtLossFraction.* The fish's weight is multiplied by 1-*fishSpawnWtLossFraction.* As we assume semelparous spawners, the exact weight loss does not have to be realistic as long as it makes fish die from poor condition. We therefore set the parameter value to 0.4 for both species.

#### Identification of potential destination cells

**Fig. 15:**
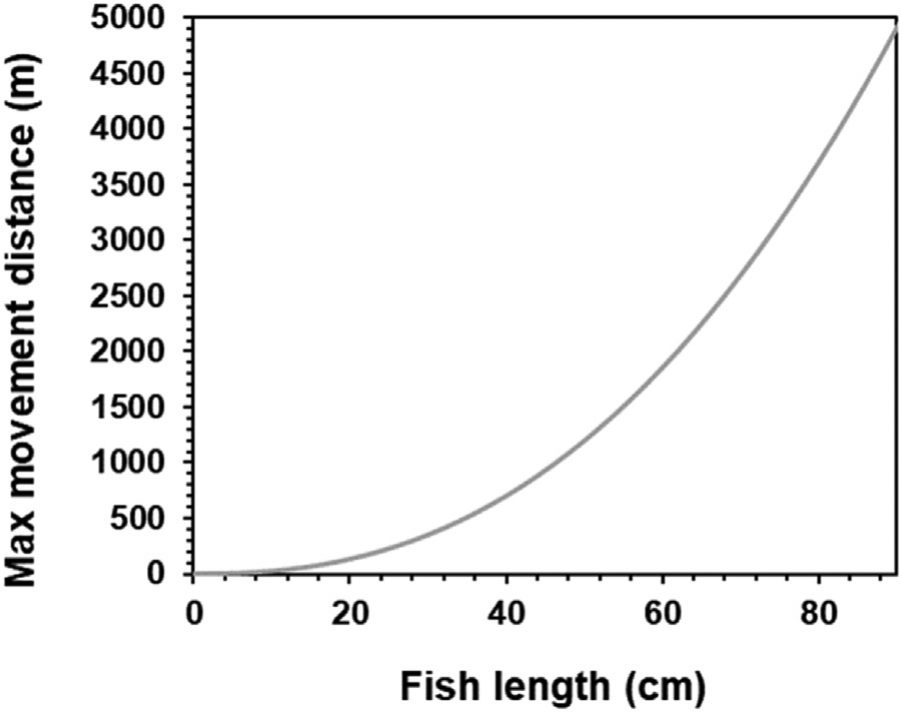
The radius of cells that a fish can move to increases with its length. Note that the maximum movement distance is calculated in cm in inSTREAM; meters are here used just for display.

To reflect different movement capabilities at different sizes, fish can only select habitat cells within a distance defined by the parameters *fishMoveDistParamA* and *fishMoveDistParamB.* We estimated parameter values so that even small fish could access most of the Gullspång rapids (Table 10; Fig. 15).

**Table 10:** Parameter values for the distance limitation for fish movement.

Parameter	Value
fishMoveDistParamA	10
fishMoveDistParamB	2.4

#### Calculation of Expected Reproductive Maturity

We used the suggested value in Railsback et al. [72] for the parameter *fishFitnessHorizon*, i.e., 90 days.

#### Length-weight relationship

The formula for calculating the length-weight relationship of a healthy juvenile fish includes the two parameters fishWeightParamA and fishWeightParamB:$\;fishWeight=fishWeightParamA\times fishLength^{fishWeightParamB}.$ Railsback et al. [70] recommended that site-specific data are used to set the length-weight parameters, as the length-weight relationships can vary among salmonids. These parameters should be fitted to length and weight data from juveniles, which in many cases are routinely collected during electrofishing. We fitted data to the formula to estimate parameters A and B for both species. Since the growth formulation is most relevant for juveniles, we collected biometric data on 0+ and 1+ fish at electrofishing 18-20 September 2018. Previous electrofishing attempts had not measured wet weight, only length. In collaboration with the electrofishing management group, we sampled fork length to the nearest mm, and wet mass on to the nearest 0.1 gram on sedated fish. We did not use weight data from individuals with uncertain species identification (i.e., suspected hybrids) for the parameter fitting.

**Fig. 16:**
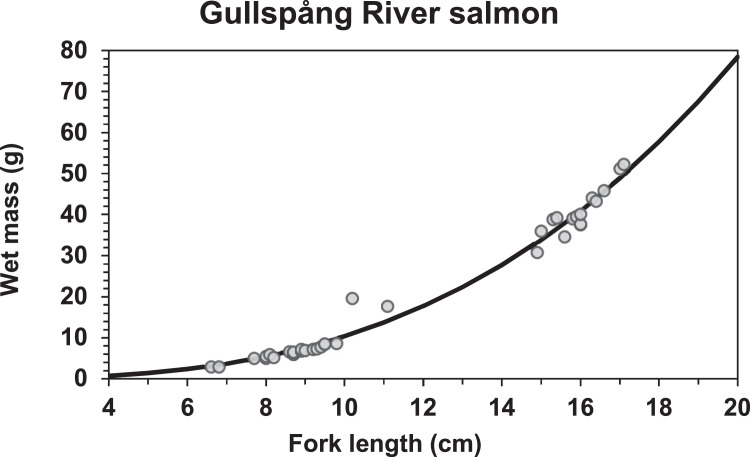
Length-weight relationship (black line) fitted to the 70% of the juvenile salmon in the best condition (grey points). Note that the x-axis is zoomed in on the range of possible fish lengths in September.

**Fig. 17:**
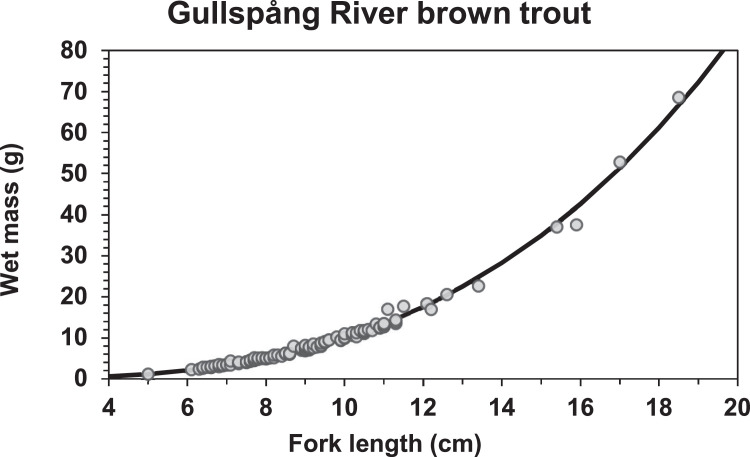
Length-weight relationship (black line) fitted to the 70% juvenile brown trout in the best condition (grey points). Note that the x-axis is zoomed in on the range of possible fish lengths in September.

We fitted the parameters by:

(1) Setting the brown trout parameter values in Railsback et al. [70] table 8, p.57 as tentative values for A and B.
(2) Calculating for each fish (N trout = 184; N salmon = 54) the expected weight based on its length and the tentative parameter values, using the formula above.
(3) Calculating a condition factor for each fish:
  $$fishCondition=\frac{fishWeight\left(obs\right)}{\left(fishWeightParamA\times fishLength^{fishWeightParamB}\right)}$$
(4) Discarding the 30% of the samples with the lowest calculated condition factor (i.e. 16 records of salmon and 55 records of trout). We discarded these data because *fishWeight* should represent fish in good condition.
(5) Calculating on the remaining records the sum of squared errors (SSE) and using Excel Solver to find the parameter value combination minimizing SSE, using the GRG Nonlinear method. The estimated parameter value pair gave a length-weight curve that was a good fit with observed data (Fig. 16; Fig. 17; Table 11).

**Table 11:** Site- and species-specific parameter values in the length-weight formula.

Parameter	Value trout	Value salmon
fishWeightParamA	0.008476	0.012784
fishWeightParamB	3.073956	2.911403

#### Drift foraging

Fish in inSTREAM can select between two modes of foraging: stationary drift-feeding and opportunistic searching for benthic or surface-oriented food. There is a range of parameters describing the drift-feeding tactic. The concentration of drifting food (in g/cm^3^) itself is given by the reach input data *habDriftConc and* is known to have a strong effect on the growth of all age classes of fish [70]. As we do not have time series data of drift in the Gullspång River, the *habDriftConc* was calibrated (section ``Calibration of mean length of 0+ fish''). Drift-feeding fish capture prey within a capture area geometry calculated from their detection distance *detectDistance.* The detection distance is determined by the light conditions (day/night) given by the *fishReactDistNightFactor,* the fish length, and the parameters *fishDetectDistanceParamA* and *fishDetectDistParamB*. Furthermore, whether a prey within a fish’ capture area is caught or not is determined by the capture success parameters *fishCaptureParam1* and *fishCaptureParam9.* We used the recommended values from Railsback et al. [72] for all drift foraging parameters (Table 12). As turbidity is zero in our model application, parameters concerning turbidity's effect on drift foraging success was ignored.

**Table 12:** Suggested drift foraging parameters from Railsback et al. [72].

Parameter	Value
fishDetectDistanceParamA	4 cm
fishDetectDistanceParamB	2
fishReactDistNightFactor	0.5
fishCaptureParam1	1.6
fishCaptureParam9	0.5

#### Active search for food

The production of benthic and surface prey, measured in g/h-cm^2^, is given by the habitat parameter *habSearchProd* (section ``Reach specific parameters'')*.* This parameter was calibrated along with the drift food concentration in section ``Calibration of mean length of 0+ fish''. We kept the other parameter values suggested by Railsback et al. [72], i.e. that a fish consumes search food production within 2 m^2^, and that a fish reduces their search food intake by 50% at night (Table 13).

**Table 13:** Search food production parameters from Railsback et al. [72].

Parameter	Value
fishSearchNightFactor	0.5
fishSearchArea	20000 cm^2^

#### Maximum consumption

We used the suggested values in Railsback et al. [72] for the allometric maximum consumption function and temperature maximum consumption function (Table 14).

**Table 14:** Parameter values from Railsback et al. [72].

Parameter	Value
fishCmaxParamA	0.628
fishCmaxParamB	-0.3
fishCmaxTempT1	0°C
fishCmaxTempT2	2°C
fishCmaxTempT3	10°C
fishCmaxTempT4	22°C
fishCmaxTempT5	23°C
fishCmaxTempT6	25°C
fishCmaxTempT7	100°C
fishCmaxTempF1	0.05
fishCmaxTempF2	0.05
fishCmaxTempF3	0.5
fishCmaxTempF4	1.0
fishCmaxTempF5	0.8
fishCmaxTempF6	0
fishCmaxTempF7	0

#### Cost of standard and activity respiration

We used the suggested values in Railsback et al. [72] for describing the bioenergetic costs of standard respiration and activity respiration (Table 15).

**Table 15:** Parameter values for respiration costs in Railsback et al. [72].

Parameter	Value
fishRespParamA	30
fishRespParamB	0.784
fishRespParamC	0.0693°C^−1^
fishRespParamD	0.03 s/cm

#### Energy intake and growth

Consumed food is converted into energy and growth using the conversion habitat parameter *habPreyEnergyDensity* (section ``Reach specific parameters'') and the energy conversion factor, the *fishEnergyDensity*. We used 5900 j/g as the parameter value for *fishEnergyDensity*, as suggested by Railsback et al. [72].

#### Fish survival from high temperature

As in real ecosystems, salmon and trout are in inSTREAM subject to a range of mortality factors that vary spatially and temporally. High stream temperatures are one of the factors that vary temporally, but not spatially as we use one temperature for the whole reach. InSTREAM model high temperature survival of salmon and trout as daily survival probabilities, with a function that has two points of interest: *mortFishHiTT1* –the temperature for which high temperature survival is 10%, and *mortFishHiTT9* –the temperature for which high temperature survival is 90%. We could not find relevant studies on longer-term (daily) survival of high temperatures for Atlantic salmon and brown trout, so we used the suggested parameter values from Railsback & Sheppard [73] (Table 16). As the discharge to the G-Rapids is from below-surface water, the water temperature rarely exceeds a level that is problematic for salmon and trout.

**Table 16:** High temperature survival values in Railsback and Sheppard [73].

Parameter	Value
mortFishHiTT1	30°C
mortFishHiTT9	25.8°C

#### Fish survival from high velocity

Due to lack of site- and species-specific data, we used the default values in Railsback & Sheppard [73] to describe high velocity related mortality (Table 17).

**Table 17:** Parameters on high velocity survival with values from Railsback & Sheppard [73].

Parameter	Value
fishMaxSwimParamA	2.8s^−1^
fishMaxSwimParamB	21cm-s^−1^
fishMaxSwimParamC	-0.0029°C^−2^
fishMaxSwimParamD	0.084°C^−1^
fishMaxSwimParamE	0.37
mortFishVelocityV1	1.8
mortFishVelocityV9	1.4
mortFishVelocityCoverFactor	0.75

#### Fish survival from stranding

Stranding of fish, i.e., when a fish is trapped in shallow water, is not likely to be an important mortality factor in the G-Rapids due to the stable flow conditions (section ``What are the effects of increasing the minimum flow?''). We therefore used the parameter values from Railsback & Sheppard [73] for stranding mortality (Table 18).

**Table 18:** Parameter values for stranding mortality, taken from Railsback & Sheppard [73].

Parameter	Value
mortFishStrandD1	-0.3
mortFishStrandD9	0.3

#### Fish survival from aquatic predation

Aquatic predation survival probability for a given fish at a given time and location is a function of the minimum predation survival of day or night, and the maximum values of a number of survival increase functions. Juvenile salmon and trout in the G-Rapids face minimal risk of predation from other fish due to the isolation of the rapids. The G-Rapids can only be accessed from a pool and weir fishway with high water velocities, or downstream from the spill gates of the dam. As the potential predatory fish species in our system are relatively weak swimmers, we have reason to believe that the fishway effectively stops their upward migration. Downstream passage through the spill gates has been observed, but we considered this a rare event. Anglers occasionally catch perch (*Perca fluviatilis*)*,* in the deep pool immediately below the dam (Robert Skogh, pers. comm.). As previously described, electrofishing data confirms the presence of the occasional predatory fish (section ``Presence of other species'').

To facilitate a possible expansion of inSTREAM for the whole Gullspång River, we parameterized the survival increase functions assuming pike (*Esox lucius*) is the main aquatic predator in the river. Pike was the most abundant predatory fish in the electrofishing records for the whole river, followed by burbot (*Lota lota*) [78]. We assume that predation risk is equal for salmon and trout when all else is equal, i.e., the predator does not have a preferred species. Note that while the resident trout form of inSTREAM 6.1 include piscivorous trout we assume that only non-salmonid fish are predators on salmon and trout in the Gullspång River. As a rule of thumb, freshwater resident brown trout become piscivorous when reaching 20 cm [43].

##### Minimum aquatic predation survival during day and night

InSTREAM-SD introduced the concept of different minimum predation survival in daytime and nighttime. In applications where larger trout were the main aquatic predators, minimum predation survival was set to be lower at night than at in daytime [73]. This is not the case in our application. We assume that aquatic predation survival will be slightly higher at night than in the day with pike as the main offender. Pike are highly visually guided predators with sensitive lateral lines [27], but with an overall lower strike and capture success when visibility is low [22]. To reflect the low density of aquatic predators, we set high values for the minimum aquatic predation survival parameters; *habFishMortAqPredNightMin* were set at 0.99 and *habFishMortAqPredDayMin* at 0.98. This means that a fish experience a daily baseline probability of 1% and 2% of dying from an aquatic predator during night and day, respectively.

##### Density of predatory trout is zero

As explained earlier, we assume that trout and salmon are not cannibalistic in this system. We therefore set parameter values to make them inactive as piscivored by giving the *fishPiscivoryLength*—the length at which the salmon and trout in the model become piscivorous —a value of 100 cm. As model fish will never reach lengths above 100 cm, the parameters *mortFishAqPredP9* and *mortFishAqPredP1* were not used.

##### Depth survival increase

**Fig. 18:**
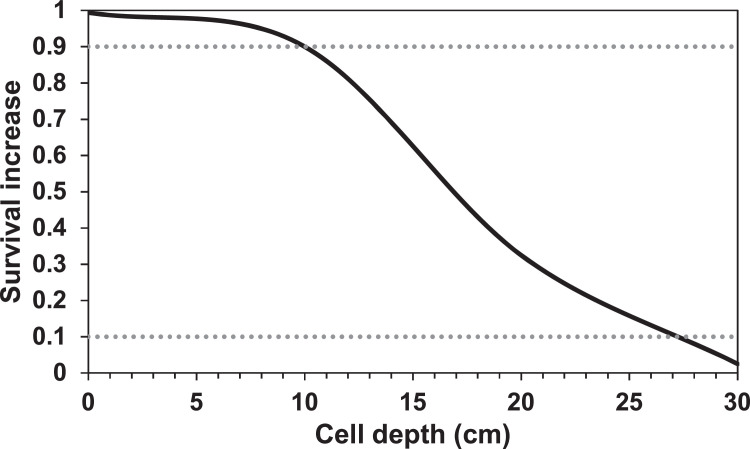
Depth survival increase function in the aquatic predation method.

Shallow water offers a survival increase from aquatic predation. This is included in inSTREAM because large predatory fish either are physically excluded, or behaviorally excluded from shallow habitat due to own predation risk. We estimated a daily survival increase of 10% for fish in cells with 27 cm water depth and 90% for fish in cells with 10 cm water depth (Fig. 18; Table 19).

**Table 19:** Parameters relating to survival from aquatic predation.

Parameter	Value
habMortFishAqPredDayMin	0.98
habMortFishAqPredNightMin	0.99
fishPiscivoryLength	100 cm
mortFishAqPredD1	27 cm
mortFishAqPredD9	10 cm
mortFishAqPredL1	12 cm
mortFishAqPredL9	20 cm
mortFishAqPredT1	8°C
mortFishAqPredT9	2°C
mortFishAqPredCoverFactor	0.95

##### Fish length survival increase

**Fig. 19:**
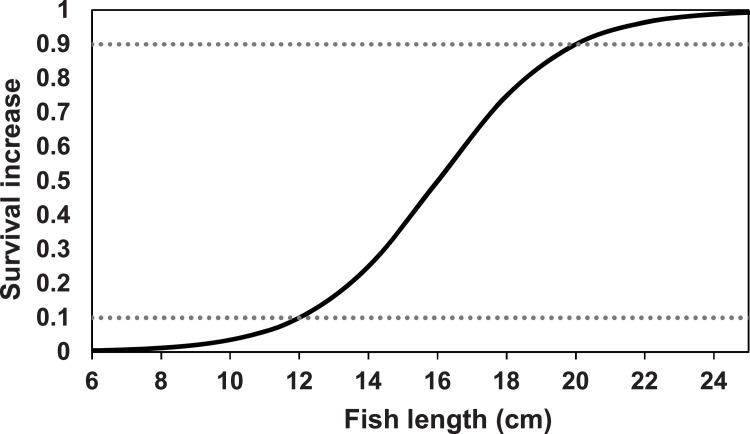
Fish length-based survival increase function.

Larger salmon and trout are to some degree protected against predation by fish from their own size. Pike is a gape-size limited predator, and the gape size of pike is linearly correlated with its body length [53]. The body depth of prey is however the main limiting measure [22]. To their disadvantage when facing a pike, salmon and trout have streamlined bodies without any defense structures. Pike caught in the Gullspång River during electrofishing within the model period (2008-2018) was between 20 cm and 45 cm [78]. Based on that size range, we estimated a daily survival increase of 10% for 12 cm fish and 90% for 20 cm fish (Fig. 19; Table 19).

##### Low temperature survival increase

**Fig. 20:**
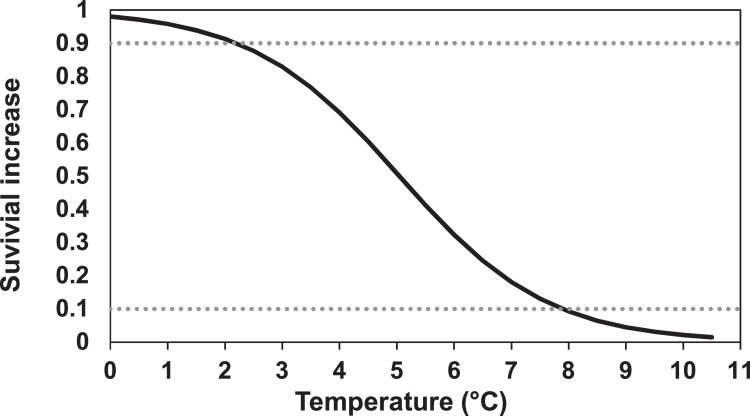
Temperature survival increase function for pike predation survival.

Adult pike has an optimal temperature for growth at about 20°C [11], which is slightly higher than that of salmon and trout. Öhlund et al. [55] studied pike attack rate on brown trout in laboratory conditions under varying temperatures, and found a sharp decline in attack rates in temperatures under 11°C. The survival increase function should therefore approach zero when temperature approaches 11°C, and likewise approach one when the temperature approaches zero. With these constraints, we approximated the values of parameters to the nearest half degree by logistic fitting (Fig. 20). The daily survival increase was set at 90% at 2°C water temperature and 10% at 8°C (Table 19).

##### Turbidity survival increase

Experiments with pike foraging under different turbidity regimes has highlighted a complex response; although pike have a shorter detection distance at high turbidity it might compensate with more frequent attacks against prey [27]. For our application where turbidity is set to zero, we ignored the turbidity parameters *mortFishAqPredU1 and mortFishAqPredU9*.

##### Cover

A fish using hiding cover has a survival increase from aquatic predation corresponding to the value of *mortFishAqPredCoverFactor*. We used the recommended value in Railsback and Sheppard [73] (Table 19).

#### Fish survival from terrestrial predation

Predation by terrestrial animals, mainly birds, is presumably a prominent source of mortality to fish in the Gullspång River. We adjusted the terrestrial predation parameter values to reflect predators observed in the Gullspång Rapids. We used the Swedish citizen science species observation database the Species Portal (Artportalen: www.artportalen.se) and own observations to get a picture of the potential terrestrial predators present in and around the G- Rapids. Table 20 lists potential salmonid-eating species sighted in the G- Rapids between 2000 and 2019 reported to the Species Portal. Vagrant individuals accompany several resident pairs of white-throated dipper (*Cinclus cinclus*) during salmon and trout spawning, when these birds probably feed on stray eggs (Robert Skogh, pers.comm.). Dippers can also feed on small salmon and trout fry, but this is unlikely to be of such a scale that it has an adverse effect on salmonid stocks in natural populations [83]. The common goldeneye (*Bucephala clangula*) is occasionally observed swimming in the rapids, but it is not known whether they target mainly fish fry or invertebrate prey. The Eurasian kingfisher (*Alcedi atthis*) has been found to prey on salmonids (e.g., [85]), but this species is a rare visitor in the G-Rapids. We consider grey heron (*Ardea cinerea*), goosander (*Mergus merganser*) and cormorant (*Phalacrocorax carbo*) to be the most important terrestrial predators on salmon and trout in the G-Rapids. The invasive American mink *Neovison vison* might also be a threat. All but one of these predators forage by diving underwater, which influences the parameterization of the survival increase functions.

**Table 20:** Species that are likely predators on salmon and trout in the Gullspång rapids, sorted in descending order after the number of observations in the open species observation database Artportalen (www.artportalen.se) 2000-2019.

Species	Sightings	Foraging tactic
White-throated dipper	Resident	Dive
Common goldeneye	Occasionally breeding	Dive
Grey heron	Common forager	Wade
Goosander	Occasional forager	Dive
Cormorant	Occasional forager	Dive
Eurasian kingfisher	Rare forager	Dive
American mink	Occasional (own obs.)	Dive

##### Minimum terrestrial predation survival day and night

All the bird predators are daytime foragers, while mink can also be night-active [87]. We therefore assumed a minimum daily terrestrial predation survival *mortFishTerr-PredNightMin* of 0.99 during the nighttime (Table 21). The daytime minimum terrestrial predation survival *mortFishTerrPredDayMin* was calibrated (section ``Calibration of mean length of 0+ fish'').

**Table 21:** Parameters and values for terrestrial predation survival.

Parameter	Value
mortFishTerrPredNightMin	0.99
mortFishTerrPredDayMin	0.9775 (section ``Calibration of mean length of 0+ fish'')
mortFishTerrPredD1	50 cm
mortFishTerrPredD9	200 cm
mortFishTerrPredL1	30 cm
mortFishTerrPredL9	50 cm
mortFishTerrPredV1	50 cm/s
mortFishTerrPredV9	150 cm/s
mortFishTerrPredH1	500 cm
mortFishTerrPredH9	-100 cm
mortFishTerrPredCoverFactor	0.95

##### Depth survival increase

**Fig. 21:**
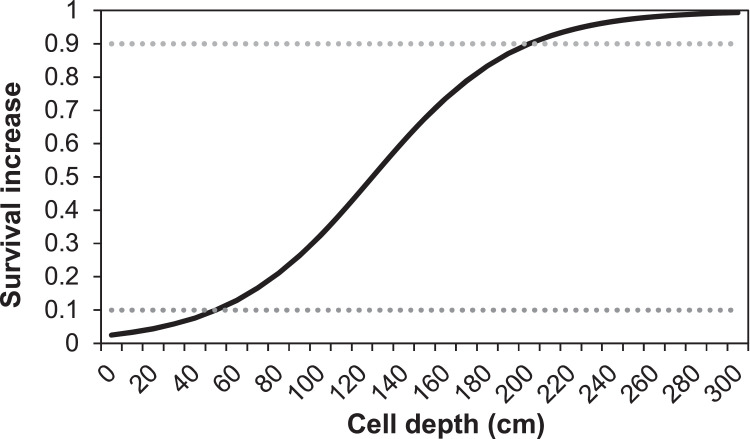
Survival increase function (black line) for cell depth in terrestrial predation, and the parameters indicating 10% survival increase and 90% survival increase.

Fish can be harder to detect by visually guided terrestrial predators when they are in a deep cell, although the predators hunt by diving (Table 20). InSTREAM includes a logistic survival increase curve that increases with increasing depths. In our hydraulic model simulations, the maximum depth in the G-Rapids varied between 2.5 m at the lowest simulated flow (0.5 m^3^/s), to 3.5 m at the highest simulated flow (30 m^3^/s). Based on the depth distribution in this reach and parameter estimates from earlier inSTREAM applications ([73] p.85), we estimated 50 cm and 200 cm for *mortFishTerrPredD1* and *mortFish-TerrPredD9*, respectively (Fig. 21; Table 21).

##### Turbidity survival increase

As turbidity is always zero in our application, the survival increase function for turbidity is not applicable.

##### Fish length survival increase

**Fig. 22:**
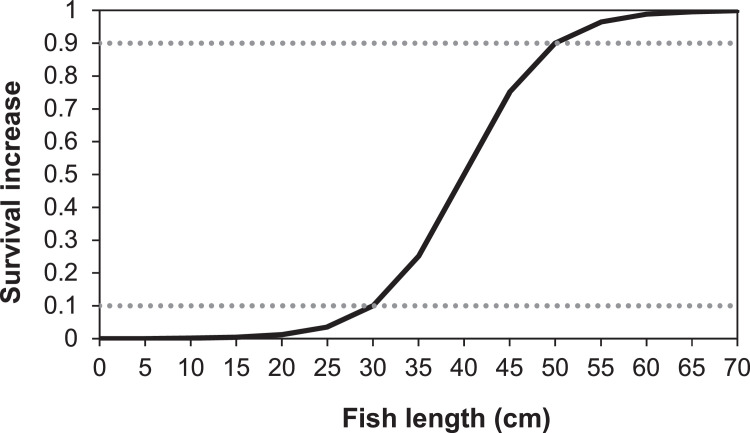
Survival increase function describing how a fish's length provides increased survival from terrestrial predators.

The group of potential fish predators (Table 20) are in the size range of ∼30 g (kingfisher) to several kg (cormorant), so it is likely that terrestrial predators predate on a wide size range of fish. Adult fish on the other hand, are likely somewhat protected from these predators due to their large size. New to this application is therefore our assumption that only very large fish have a size benefit when it comes to surviving terrestrial predation. We therefore set the terrestrial predation survival increase function with 30 cm and 50 cm as *mortFishTerrPredL1* and *mortFishTerrPredL9*, respectively. With these parameter values we avoided the unrealistic initial model behavior that adults >50 cm regularly died from terrestrial predation (Fig. 22; Table 21).

##### Water velocity

**Fig. 23:**
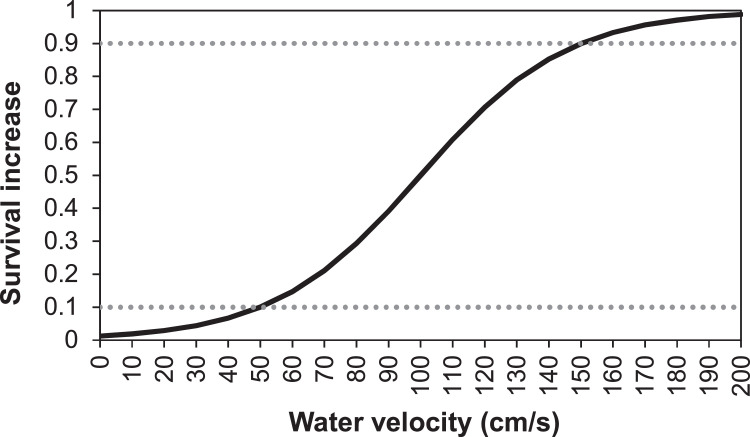
Survival increase function describing how water velocity is related to higher survival from terrestrial predators.

Higher water velocities offer protection from terrestrial predators, for instance through impaired detectability from the air when the water surface rifles. This effect is incorporated as a terrestrial predation survival increase function that increases with increasing water velocities. We estimate that 50 cm/s and 150 cm/s are reasonable values for *mortFishTerrPredV1* and *mortFishTerrPredV9* (Fig. 23; Table 21).

##### Distance to hiding cover and cover factor

**Fig. 24:**
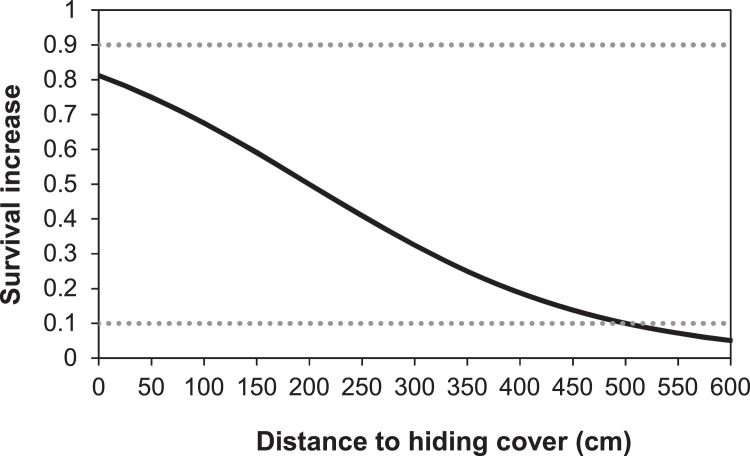
Survival increase function describing how distance to hiding cover provides increased survival probability from terrestrial predation.

Fish that are feeding close to hiding cover have the possibility to hide when they detect a predator. This possibility is included as a survival increase function of distance to hiding cover, given by the parameters *mortFishTerrPredH1* and *mortFishTerrPredH9* (Fig. 24). We used the suggested values in Railsback & Sheppard [73]. Furthermore, fish that are hiding have a survival increase value given by the parameter *mortFishTerrPredCoverFactor*. For this, we kept the parameter value estimated by Railsback & Sheppard [73] (Table 21).

#### Out-migration

Atlantic salmon and brown trout juveniles both migrate from the G-Rapids downstream to Lake Vänern and stay there until they reach maturity. The outmigration process likely resembles that of anadromous Atlantic salmon and sea trout, with the exception that fish do not need to adapt physiologically to salt water. We have deliberately called this “out-migrants” and “out-migration” instead of “smolt” and “smolt migration”. To our knowledge, there have been no direct studies on juvenile out-migration in the Gullspång River. We have therefore not restricted out-migration temporally the model, but rather kept the possibility open for year-round out-migration. This would correspond to both parr, presmolt and smolt out-migration, all of which have been observed in river systems with year-round out-migration surveillance [1,46].

Runnström [76] performed studies on salmon and trout in Lake Vänern based on scale samples collected from adult fish (9 salmon and 11 brown trout) caught in the mouth of the Gullspång River on their spawning migrations 1912-1921. Based on scale readings and size back-calculations, he concluded that trout generally were larger than salmon at out-migration, with a mean length of 27 cm for trout versus 24 cm for salmon (Table 22). He also found that juvenile salmon and trout out-migrated during their 2^nd^, 3^rd^ and 4^th^ calendar year. A more recent study was done by Ros [75], using data from Gullspång salmon collected in the 1960’s and -70’s. It is however unclear whether he collected data directly from smolt, or if he also backtracked size and age at outmigration from scales of adults. Ros [75] found only 1^st^ and 2^nd^ calendar year out-migrants and pointed out what is seemingly a shift towards younger smolt ages for salmon between the early and latter half of the 20^th^ century. Modern electrofishing data (section ``Length distribution on electrofishing dates'') agrees with a younger and smaller size at outmigration in comparison to the limited material of Runnström [76].

**Table 22:** Historical data on Gullspång River salmon and trout out-migrants per age class. The sample size of Ros [75] is unknown.

Source	Mean length at outmigration in cm (sample size)									
	Gullspång salmon					Gullspång trout				
	1+	2+	3+	4+		1+	2+	3+	4+	
Runnström [76]	-	19.4 (2)	21.1 (3)	28.2 (4)		-	25.3 (6)	25.1 (3)	35.4 (2)	
Ros [75]	14.1	19.1	-	-		-	-	-	-	

**Fig. 25:**
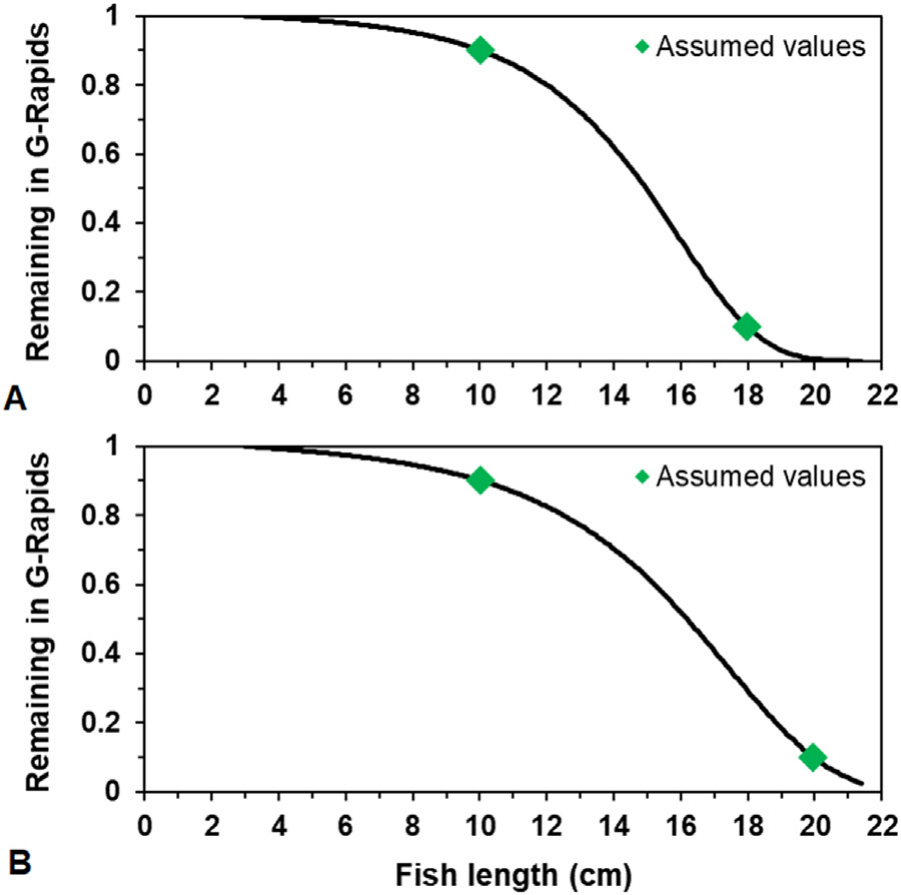
The probability that a fish remains in the G-Rapids decreases with its length. A) We assumed that at 10 cm, 90% of salmon juveniles remain in the G-Rapids, while at 18 cm only 10% remain. B) We assume that at 10 cm, 90% of trout juveniles remain in the G-Rapids, while at 20 cm only 10% of the cohort remain.

We implemented outmigration in this inSTREAM application with two added parameters: *mortFishOutmigrationL1 and mortFish-OutmigrationL9*. Outmigration is modeled as a stochastic event, with the probability of outmigration being a logistic function of fish length: juvenile salmon become more likely to migrate out as they become longer. The logistic function was parameterized to reproduce assumed probabilities of remaining in the reach (i.e., the cumulative probability of not migrating out over all time steps up to the present), at an assumed growth rate of 0.5% increase in length per day. We assumed that both species are 90% likely to remain at a length of 10 cm, brown trout are 10% likely to remain at 20 cm length and salmon are 10% likely to remain at 18 cm length (Fig. 25). To reproduce these assumed probabilities, we fitted the parameters *mortFish-OutmigrationL1* and *mortFishOutmigrationL9* (lengths at which daily probability of outmigration is 0.1 and 0.9) to a logistic function of cumulative probability of staying (see [70], p. 9) (Table 23).

**Table 23:** Estimated parameters for outmigration of juvenile fish.

Parameter	Value trout	Value salmon
mortFishOutmigrationL1	32.55 cm	28.08 cm
mortFishOutmigrationL9	21.09 cm	18.67 cm

#### Survival from poor condition

We used the parameter values from Railsback & Sheppard [73] for poor condition mortality (Table 24).

**Table 24:** Parameter values for starvation mortality in Railsback & Sheppard [73].

Parameter	Value
mortFishConditionK1	0.3
mortFishConditionK9	0.6

### Redd variables and parameters

Redds are subject to the mortality sources high temperature, low temperature, scour, superimposition and dewatering. Note that inSTREAM does not separate between the periods of egg development and of the yolk-sac alevin development in the gravel, and these are subject to the same redd mortality and development parameters.

#### Egg survival from dewatering and scouring

The parameter *mortReddDewaterSurv* quantifies the proportion of live eggs that can survive one day when the redd is dewatered. We use the suggested value from Railsback and Sheppard [73]. The parameter *mortReddScourDepth* is interpreted as the depth at which the eggs are buried in the redds. The people regularly doing redd surveys in the river report that the topmost eggs in the pockets might be buried 10-30 cm deep (Jukka Syrjänen, pers.comm.). We therefore estimate a *mortReddScourDepth* of 20 cm for both species (Table 25).

**Table 25:** Parameter values for egg survival from dewatering and scouring.

Parameter	Value
mortReddDewaterSurv	0.9
mortReddScourDepth	20 cm

#### Egg survival from high and low temperatures

The inSTREAM redd entity, i.e., eggs and alevins, also face direct temperature-induced mortality. Salmonid eggs and alevins have an optimal temperature range for survival during development [58]. If river temperature deviates far from the optimum, egg mortality may occur. In many systems, the negative effects of temperature extremes on redd survival can be mediated by spawning in areas with groundwater upwelling [31]. The low temperature tolerance of eggs is represented by the parameters *mortReddLoTT1* and *mortReddLoTT9*, while the upper temperature tolerance is represented by *mortReddHiTT1* and *mortReddHiTT9*. These parameters correspond to the temperatures at which 10% and 90% of the eggs will survive daily, respectively. We reviewed peer-reviewed literature on egg development under different temperature regimes. We translated survival over the studies’ reported developmental periods to inSTREAM's daily survival probabilities using the formula:

$$Daily\;survival=Observed\;survival\;whole\;period^{\frac{1}{length\;of\;period\;\left[days\right]}}$$

Jonsson and Jonsson [44] reported lower and upper temperature limits for survival of brown trout embryos at 0 and 14°C, and of alevins 0 and 22°C. Ojanguren & Braña [56] found that the optimal temperature for egg development in a Spanish population of brown trout was between 8 and 10°C. They also found declining survival at both higher and lower temperatures, with all eggs dying before hatching when temperatures exceeded 16°C. Other studies reported an optimum temperature range over which the highest proportion of embryos hatch; for brown trout this interval is shown to be between 1 and 8°C [44].

**Fig. 26:**
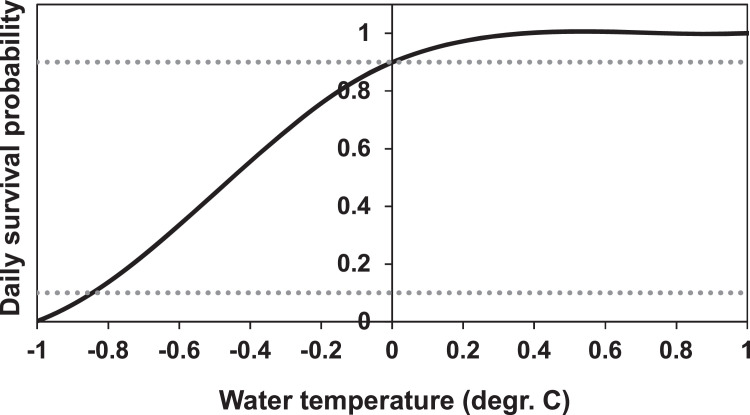
Daily survival of eggs at low temperatures estimated from literature values, mainly in Wallace and Heggberget [86], gives a 10% daily survival probability at -0.8°C and 90% daily survival probability at 0°C.

Egg development in brown trout and Atlantic salmon has been thought to cease at 0.5°C [44]. Nevertheless, Wallace and Heggberget [86] managed to hatch Atlantic salmon eggs that were kept in tanks with an average temperature of 0.17°C with high survival (80-90%). We did not find any studies that had tried to incubate brown trout eggs in temperatures < 4°C and reported mortality. Ojanguren and Braña [56] reported a survival of 3% from fertilization to hatching of brown trout eggs at 4°C, which translates into a daily survival probability if 0.991. In comparison, Jungwirth and Winkler [47] found a survival of 78% at 4°C for brown trout in Maine, U.S.A., which translates into a daily survival probability of 0.998. Relying mainly on the study by Wallace and Heggberget [86], we assume a daily survival of 0.999 for Atlantic salmon eggs at 0.5°C, and a daily survival of 0.9 at 0°C. As we have no data on brown trout below 4°C, we assume the same low temperature survival for both species (Fig. 26; Table 26).

**Table 26:** Parameter values describing egg mortality under high and low water temperatures.

Parameter	Value
mortReddHiTT1	19°C
mortReddHiTT9	15°C
mortReddLoTT1	-0.8°C
mortReddLoTT9	0°C

**Fig. 27:**
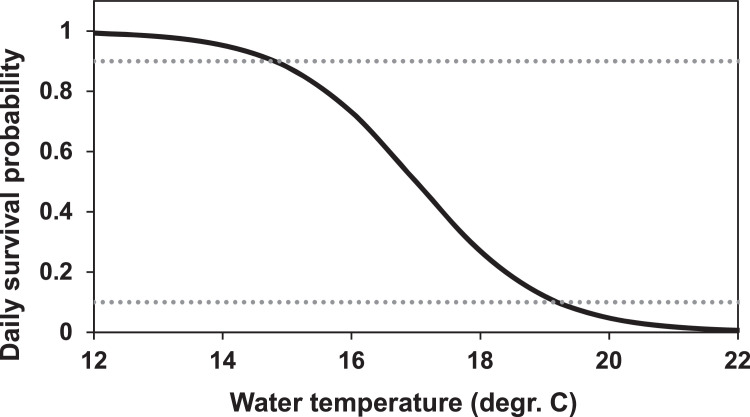
Daily survival of eggs at high temperatures estimated from literature values gives a 90% daily survival probability at 15°C and 10% daily survival probability at 19°C.

Jungwirth and Winkler [47] also incubated brown trout eggs up to 16°C, while Ojanguren and Braña [56] incubated brown trout eggs at up to 18°C. From Jungwirth and Winkler's data, daily survival dropped from 0.984 at 12°C to 0.882 at 14°C, while at 16°C none of the eggs hatched. Likewise, in Ojanguren & Braña's study the daily survival at 13.9°C was 0.96, while all the eggs died before hatching at 16°C and 18°C. It was not reported exactly how long it took for all the eggs to die at the uppermost temperatures in either of the studies, so the daily survival remains unknown. From Gunnes [36], the daily survival of Atlantic salmon incubated at 12°C was 0.984. For high temperature survival, we decided to rely more on the more recent study by Ojanguren and Braña [56], and assume a daily survival of 0.95 at 14°C and 0.5 at 17°C. We assume the same high temperature survival in both species (Fig. 27; Table 26).

#### Egg development

The rate of embryo development in salmonids is a temperature-dependent process (e.g., [26,58]). There is a non-linear relationship between water temperature and total time of development from fertilized eggs to hatching yolk-sac alevins to emerging fry. Therefore, inSTREAM uses a daily fractional egg development formulation incorporating reach temperature, and three redd development parameters. The redd state variable *fracDeveloped* (0-1) is updated daily by adding the value of *reddDailyDevel* as: $reddDailyDevel=reddDevelParamA+reddDevelParamB\times T+reddDevelParamC\times T^{2}$. Fry emerge from the gravel when *fracDeveloped* ≥ 1. The formula is also referred to as the “log inverse Belehrádek model” (e.g., [8]) or the as “the power law with temperature correction” (e.g. [14]). It is one of several equations proposed for fitting the time-temperature relationship to experimental data of eggs incubated at near constant temperatures.

**Fig. 28:**
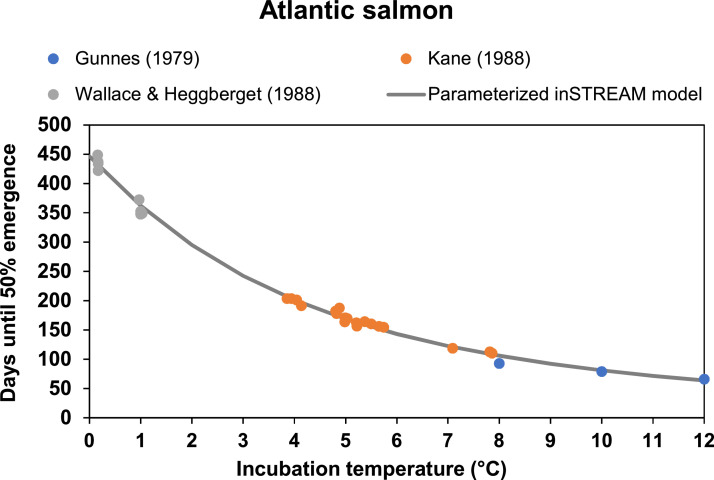
Mathematical relationship between incubation temperature and days until 50% emergence for Atlantic salmon.

**Fig. 29:**
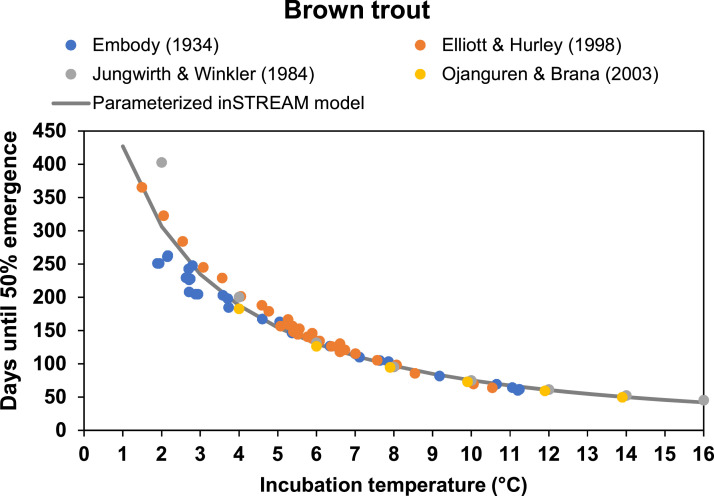
Mathematical relationship between incubation temperature and days until 50% emergence for brown trout.

**Fig. 30:**
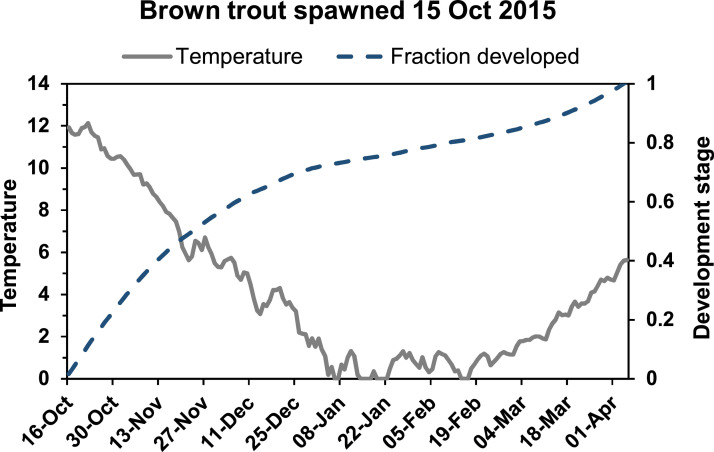
Example redd (=eggs and alevins) development for a hypothetical brown trout that spawned on 15 October 2015.

The relationship between temperature and egg development in salmonids has been subject to scientific inquiry since at least the early 20^th^ century. Unfortunately, many of these incubation studies have not published raw data, but rather report aggregated data, figures, or other mathematical formulas. Moreover, many studies reports number of days until 50% of the eggs have hatched per incubation temperature, and not until 50% of the fry have emerged. We included these data by assuming a linear relationship between hatching date and emergence date for static incubation temperatures. Kane [48] noted that in his study on Atlantic salmon incubation, the time from fertilization until 90% hatching constituted on average 58.3% of the total number of days, from fertilization to initial feeding. This was supported by Crisp [13], who noted a linear relationship that was very similar for a range of salmonid species. We therefore included studies that report time to 50% hatching by estimating time to 50% emergence using the regression formula by Crisp [13]: Daysuntilemergence=1.66×Daysuntilhatching+5.4. We extracted data points from tables and from figures in three papers for Atlantic salmon (Norway: [36]; USA: [48]; Norway: [86]), and four papers for brown trout (England: [25]; USA: [26]; Austria: [47]; Spain: [56]). The number of data points reflects the sample sizes in the respective studies and did not apply meta-analytical tools such as weighing of the studies. The parameter fitting was done by minimizing the residual sum of squares between model and literature data points using MS Excel Solver (Fig. 28; Fig. 29; Table 27). As an example, using these parameter values, a brown trout spawning on 15 October 2015 would emerging from 5 April 2016 according to our temperature time-series (section ``Reach temperature'') (Fig. 30).

**Table 27:** Parameter values in the egg development formula for brown trout and Atlantic salmon.

Parameter	Value trout	Value salmon
reddDevelParamA	0.001492714	0.002239196
reddDevelParamB	0.000813082	0.00046686
reddDevelParamC	0.000036268	0.00005409

Overall, there were surprisingly few studies reporting duration of development per incubation temperature relationship for Atlantic salmon and brown trout. We also did not find any experimental incubation studies comparing Atlantic salmon and brown trout development under identical laboratory conditions. There is also likely to be a genetic component to the temperature-development relationship, driven by adaptation to varying climatic conditions in different salmonid species’ ranges [42]. As river temperatures are expected to increase with climate change, an understanding such species-specific temperature relationships will be of increasing importance [24,45].

#### Creation of fish from eggs

In nature, salmonid fry emerge from the gravel over several days. This phenomenon is also represented in an inSTREAM submodel. When fracDeveloped is ≥1, remaining eggs will turn into new fish over the course of ten days. This transition is carried out by having 10% of the eggs turn to fish the first day, 20% of remaining eggs to fish on day two etc. Emerging fish are aggregated into superindividual objects.

Model fish are created either from the initial population file, the file of arriving adults ready to spawn, or from successfully developed eggs. New fish created from eggs are assigned the species of and placed in the same habitat cell as its redd and assigned sex randomly (with a 50:50 probability). The length of a newly emerged fish is assigned from a distribution given by the parameters *reddNewLengthMean* and *reddNewLengthStdDev*. The new fish are further assigned a weight from the weight-length formula (section ``Length weight relationship''). Literature on emerging Atlantic salmon and brown trout fry often reported mass, not length, but Bror Jonsson (pers.comm.) suggested that 2 cm was a reasonable mean length for both brown trout and Atlantic salmon. We assumed a standard deviation of 0.2 cm in lengths of emerging fish (Table 28).

**Table 28:** Parameters for size distribution of new fish

Parameter	Value
reddNewLengthMean	2 cm
reddNewLengthStdDev	0.2 cm

### Initial population

We set the initial fish population in inSTREAM based on data from the 106 fish electrofished in the G-Rapids in 2008 [78]. As only a proportion of the reach was fished, this initial population is likely a gross underestimation of the number of fish in the reach. We chose to use the data because inSTREAM needs to be initialized with a starting population, and electrofishing data provides a baseline of what we know. For each species’ input file, we copied the initial abundance, and calculated mean length, standard deviation of length for each age class of each species (Table 29; Table 30).

**Table 29:** The initial population of brown trout in the G-Rapids.

Date(Y-M-D)	Age	Number	Mean length (cm)	SD length (cm)
2018-09-20	0	29	10.4	1.6
2018-09-20	1	3	15	0.9

**Table 30:** The initial population of Atlantic salmon in the G-Rapids.

Date(Y-M-D)	Age	Number	Mean length (cm)	SD length (cm)
2018-09-20	0	74	7.9	1.0
2018-09-20	1	0	12	1

## Conceptual model evaluation

This TRACE element provides supporting information on the simplifying assumptions underlying a model's design, both with regard to empirical knowledge and general, basic principles.

InSTREAM has been scrutinized over more than twenty years of model development. Railsback & Harvey [68] showed that the theory used by inSTREAM to represent the primary adaptive behavior of fish, i.e., habitat selection, could reproduce six observed patterns in how salmonids adapt in terms of habitat selection in response to factors affecting growth and predation risk. This theory was generalized by Railsback and Harvey [66] into a broad representation of adaptive tradeoff decisions in individual-based models. Furthermore, Railsback et al. [71] showed that inSTREAM reproduced five diverse population-level patterns observed in real trout. The ability of inSTREAM 6 to represent the second key adaptive behavior, the choice between feeding vs. hiding during the day and at night, was validated by Railsback et al. [69]. Through simulation experiments, they reproduced eight observed patterns in how this behavior responds to various environmental and competitive conditions. Moreover, Harvey et al. [40] showed that inSTREAM version 6 could predict the observed difference in trout biomass between sites above and below a flow diversion, in a small stream. Lastly, Harvey and Railsback [38] showed that inSTREAM accurately predicted effects of flow diversion on trout growth in a controlled experiment.

## Implementation verification

This TRACE element provides supporting information on computer code testing and code implementation of the model.

The original inSTREAM 6.1 has been thoroughly tested by its developers using code reviews and independent re-implementation of major submodels. An archive of code tests and documentation for this modified version and earlier versions is available upon request from Steven Railsback (steve@langrailsback.com). The code changes implementing model changes to this application (described in chapter 2) were tested by examining graphical displays and file output.

The Swarm library used by inSTREAM 6.1 is no longer maintained. Version 7 of inSTREAM (https://ecomodel.humboldt.edu/instream-insalmo-current-versions), which is under development, will be implemented in the modeling environment NetLogo [89].

## Model output verification

This TRACE element provides supporting information on how well model output matches observations and how much calibration and effects of environmental drivers were involved in obtaining good fits of model output and data.

### Calibration of mean length of 0+ fish

Sensitivity analyses in earlier inSTREAM applications have shown that simulated fish size and abundance are most sensitive to food availability parameters and terrestrial predation parameters (e.g., [70]). We therefore parameterized the highly uncertain parameters *habDriftConc, habSearchProd* and *mortFishTerrPredDayMin* using inverse modeling to reproduce young of the year fish lengths from electrofishing (section ``Length distribution on electrofishing dates''; Table 31). The calibration was done by varying the parameters within the null scenario *Flow0* (section ``Reach discharge'') and with 20 returning adults per species and year (section ``Arrival of spawners to the G Rapids''). We divided the different years’ electrofishing records into initialization, calibration, and validation (Table 31). We assume that the mean length of 0+ salmon and trout from year to year are independent observations due to high inter-annual variability in environmental conditions and electrofishing day. Records from the same year were however used together (Table 31).

**Table 31:** Electrofishing data from the G-Rapids [78] divided into initialization, calibration, and validation. *Excluded due to potential bias from the extreme flood during the winter of 2011-2012.

Electrofishing date	Our usage	0+ salmon		0+ trout	
		N	Mean length	N	Mean length
2008-10-07	Init. Pop.	74	7.9 cm	40	10.4 cm
2009-09-29	Calibration	31	8.7 cm	28	10.5 cm
2010-09-28	Validation	74	7.6 cm	90	9.4 cm
2012-09-26	Not used*	23	9.2 cm	21	11.8 cm
2013-06-18	Calibration	24	4.1 cm	75	4.9 cm
2013-09-19	Calibration	34	7.3 cm	59	9.6 cm
2014-06-17	Validation	24	5.0 cm	40	5.8 cm
2014-09-24	Validation	1	6.1 cm	84	10.5 cm
2015-09-24	Calibration	59	8.7 cm	74	9.8 cm
2015-09-29	Calibration	22	9.5 cm	33	11.5 cm
2016-09-22	Validation	225	7.7 cm	137	10.6 cm
2018-09-20	Calibration	15	8.1 cm	136	8.7 cm

We compared the mean length of assumed 0+ fish at electrofishing with the average length of Age0 model fish sampled from the model on the same dates. We selected the parameter value combination that minimized the sum of squared residuals and produced model results with more than zero live 0+ fish of respective species around the time of electrofishing. We initially did a calibration within a broad range of parameter values, and subsequently narrowed it down to a finer calibration. All calibration was done with the juvenile superindividual ratio set to 10 and with one replicate per scenario. For the broad calibration, we performed a factorial experiment where we linked the parameters *habDriftConc* and *habSearchProd* as four different levels of food availability (Table 32) and combined these with three different *mortFishTerrPredDayMin* values (Table 31), which resulted in 3 × 4 = 12 scenarios.

**Table 32:** The four levels of food availability used in the initial broad calibration.

Food availability	habDriftConc =	habSearchProd =
“Low”	3.5E-10	5E-7
“Medium”	7E-10	1E-6
“High”	1.5E-9	2.5E-6
“Very high”	3E-9	5E-6

In the broad calibration, scenario VII, with high food availability and a *mortFishTerrPredDayMin=0.97,* outperformed the other scenarios in terms of residual sum of squares (RSS) (Table 33). Furthermore, there were live 0+ salmon and trout in that scenario at all calibration electrofishing dates. To improve fit to the data, we performed a calibration with parameter values around scenario VII. The food parameters were still linked, representing total food availability, with two levels above and two below the food availability in scenario VII (Table 34). We calibrated with four different levels of *mortFishTerrPredDayMin*, resulting in 4 × 4 = 16 scenarios (Table 35). The RSS between modelled and observed mean lengths were generally lower in the narrow calibration (Table 35). We chose the parameter value combination with the lowest RSS (Table 35; Table 36) for all subsequent simulation experiments.

**Table 33:** Residual sum of squares of the twelve combinations of food and predation survival, between modeled mean lengths and observed mean lengths. The combination in VII had the best fit.

RSS	mortFishTerrPredDayMin =		
**Food availability**	0.94	0.97	0.985
“Low”	254	245	233
“Medium”	137	74	58
“High”	239	51 (VII)	104
“Very high”	99	367	665

**Table 34:** Food availability levels in the second, narrow calibration around the best fit in the broad calibration (habDriftConc=1.5E-9 and habSearchProd=2.5E-6).

Food availability	habDriftConc=	habSearchProd=
“A”	1.1E-9	1.75E-6
“B”	1.3E-9	2.125E-6
“C”	1.875E-9	3.125E-6
“D”	2.25E-9	3.75E-6

**Table 35:** Residual sum of squares for the fourteen combinations of food availability and minimum predation mortality in the narrow calibration. *Best fit.

RSS	mortFishTerrPredDayMin=			
**Food availability**	0.955	0.9625	0.97375	0.9775
“A”	31	30	23	12*
“B”	17	23	13	45
“C”	41	69	106	112
“D”	87	139	187	192

**Table 36:** Calibrated parameter values for drift concentration (section ``Drift food concentration''), “search food” production (section ``Reach specific parameters'') and baseline terrestrial predation mortality in the daytime (section ``Minimum terrestrial predation survival day and night'').

Parameter	Value
habDriftConc	1.1E-9
habSearchProd	1.75E-6
mortFishTerrPredDayMin	0.9775

### Validation of predicted mean lengths of 0+ fish

We validated lengths of 0+ salmon and trout with the same method as in calibration, but with the validation part of the electrofishing dataset. We compared observed mean lengths of 0+ fish with modelled mean lengths of 0+ fish on the exact same date for the same species, for all ten replicates of the null alternative scenario (*Flow0* and 20 adults per species and year). We validated the model by visually assessing graphical output and in terms of residual sum of squares.

**Fig. 31:**
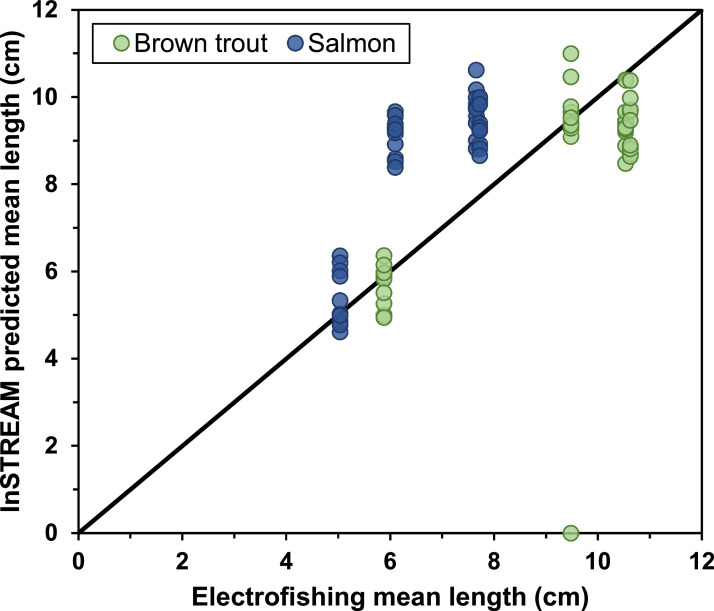
Validation of mean length produced by the model (ten model replicates, four validation dates) with respect to mean lengths at electrofishing. The black diagonal line represents 100% agreement between observed and modelled mean lengths.

The simulated mean length of 0+ fish agreed with the observed mean lengths (RRS=300; N=80) (Fig. 31). The RSS was higher due to more observations and replicates used during validation than during calibration. Generally, the lengths of salmon were overestimated, whereas the model underestimated the lengths of large brown trout. Also, note that in one of the model replicates one validation date had zero live 0+ fish. We chose not to further refine the calibration out of concern for overfitting the calibration data.

## Model analysis

This TRACE element provides supporting information on model output sensitivity to changes in model parameters (sensitivity analysis), and how well the emergence of model output has been understood.

### Sensitivity analyses

As there are a total of 137 variables in the fish parameter files, and 23 variables in the habitat parameter file, a full sensitivity analysis of our inSTREAM application is unfeasible. Albeit results can be partly site-specific, almost all of inSTREAM's parameters have been subject to sensitivity analyses in previous applications (e.g., [16,70]). We therefore focused on the new parameters in this application, as well as on the maximum temperature for spawning. The latter was found important in our application [9]. The key output evaluated were the number of large out-migrants per year, growth of 0+ and survival from fry to out-migration.

#### Spawning cell selection uncertainty fishSpawnCellUncert

In initial model runs, superimposition mortality was excessively high, resulting from an unrealistically high aggregation of redds in a few cells. The assumption that spawning females can perfectly identify the cell with the best spawning conditions led to the same cell being chosen over several consecutive time steps with the stable hydraulic conditions we used in our application. To keep unrealistically high superimposition mortality from biasing results, we reduced the adult females’ ability to sense the best spawning cell. We considered this a realistic notion, as a female salmon and trout cannot possibly have full information on what cell is the most suitable for spawning at any given time. To incorporate this change, we modified inSTREAM so that each spawning female selected randomly from among all cells with spawning habitat quality within a range of the best quality. This range was defined by the new parameter *fishCellSpawnUncert* (section ``Select spawning cell and move here''). To assess the effect of the newly introduced parameter on the key model results, we carried out a broad sensitivity analysis varying the parameter's value in five replicates. The values tested were 0.05, 0.1, 0.15 (null scenario), 0.2, 0.3, 0.4, 0.5 and 0.7. All other input was kept the same as in the null scenario.

We found that an increasing value of the *fishCellSpawnUncert* parameter led to slightly increasing egg survival, which was due to less superimposition. The model was not as sensitive to *fishSpawn-CellUncert* as initially anticipated. Each cell's *spawnQuality* (section ``Select spawning cell and move here'') is recalculated for each spawner. As discussed in section ``High temperature spawning limit fishSpawnMaxTemp'', temperature restrictions on spawning (section ``Decide whether to spawn'') delayed brown trout spawning until the last days of the spawning period.

#### Superindividual ratio juveSuperindividualRatio

The superindividual ratio is included in this obligate anadromous version of inSTREAM to increase computational speed. When there are so few fish and relatively small cell as in our application, the superindividual ratio could have an influence on model results. During length validation (section ``Validation of predicted mean lengths of 0+ fish''), we noted conspicuous deviations in mean lengths between electrofishing data and model predictions for a superindividual ratio other than used at calibration. We therefore tested the sensitivity of predicted growth to different values of the juvenile superindividual ratio parameter, *juveSuperindividualRatio*. We used the values 2, 5, 8, 10 (null scenario), 15 and 20. All other input was kept the same as in the null scenario.

We found that the predicted total number of out-migrants, standardized by total number of redds, varied irregularly with the parameter value of the juvenile superindividual ratio. Upon closer inspection, we found that the reason for the sensitivity to juvenile superindividual ratio in this application was in fact its influence on the growth submodel. The model overestimated growth when the superindividual ratio was set to 2 and 5, and underestimated growth when the superindividual ratio was set to 15 and 20. A superindividual ratio of 8 made less of a difference from a ratio of 10, the value which was initially used to calibrate 0+ lengths through the parameters drift food concentration, search food concentration and terrestrial predation. We therefore used the same superindividual ratio in the experiments as used in calibration of growth and recommend this is also done in the future.

This degree of sensitivity to low values of the juvenile superindividual ratio has not been found in sensitivity analyses for other applications (e.g., [65]), which can suggest that we in our application are approaching the lowermost limit to cell size inSTREAM. “Large” superindividual objects can deplete resources in small cells. The cell size and shape are entirely user-defined, and represents a tradeoff between computational speed, preparation time in a GIS, and hydraulic accuracy. Further studies of the scale-dependency of inSTREAM to different sized fish and different bed morphologies should conducted in the future, as part of continuous model improvement.

#### High temperature spawning limit fishSpawnMaxTemp

**Fig. 32:**
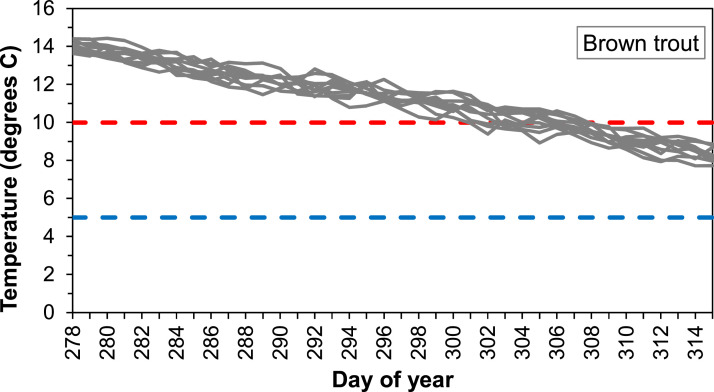
Reach temperature (grey lines) during the trout spawning period (Julian day 278- 314) of 2008-2017 and upper (red) and lower (blue) temperature criteria for spawning (dotted lines).

In our model runs, the high temperature limit *fishSpawnMaxTemp* = 10°C (section ``Decide whether to spawn'') was found to constrain brown trout spawning. This became obvious upon closer inspection of the input temperature data in relation to the spawning criteria (Fig. 32). In fact, the maximum temperature parameter resulted in brown trout being able to spawn only during the last few days of the spawning period, and, as a result, a high proportion of the redds were superimposed.

The *fishSpawnMaxTemp* parameter value was set based on the assumption that female salmon and trout will not spawn when the river temperature is outside the range of optimal egg development (section ``Decide whether to spawn''). As our temperature data (section ``Reach temperature'') is associated with high uncertainty, we could have relaxed the temperature-related spawning criteria until a more robust air-water temperature model was made. This was however discovered too late in the process for it to be feasible to change.

**Fig. 33:**
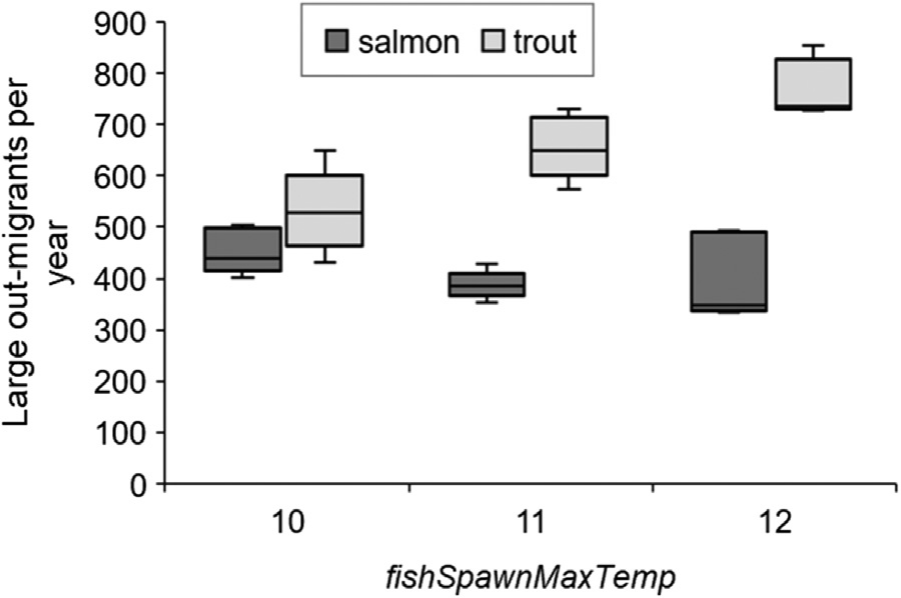
The number of large out-migrants produced annually under different assumed fishSpawnMaxTemp values.

To test the model's sensitivity to *fishSpawnMaxTemp* we ran two simulations each with five replicates, with the *fishSpawnMaxTemp* increased by one and two degrees: 11 and 12°C respectively and compared with the null scenario (10°C). We found that what we set as the maximum temperature for spawning had large effect on the simulated number of large out-migrants per year (Fig. 33). Setting the *fishSpawnMaxTemp* to 11°C or 12°C led to all females spawning but had no clear effect on egg survival. Higher values of this parameter let trout spawn earlier, which presumably caused trout to emerge earlier. Trout rearing success increased with the value of *fishSpawnMaxTemp,* causing the simulated higher production. This is in line with the conception that brown trout and salmon use temperature as a cue to spawn because of a strong selection on timing of emergence [21,45].

#### Outmigration mortFishOutmigrationL1 and –L9

**Fig. 34:**
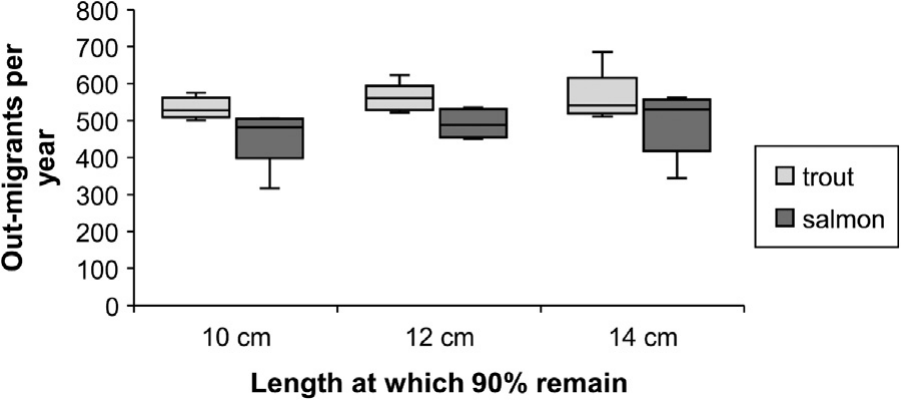
The predicted number of large out-migrants per year remained stable in the Flow0 scenario, regardless of whether we assumed that 90% of fish remained in the G-Rapids when they were 10 cm, 12 cm, or 14 cm.

The outmigration parameters are uncertain in our model application, as we to date have no data on smolt, presmolt or parr out-migration in the Gullspång River. We therefore investigated the sensitivity of the key model result (average number of large out-migrants per year) to our assumption about the fish length at which there is a 90% probability of still residing in the G-Rapids (Table 37). We assumed the same lengths at 10% probability of still residing in the rapids as earlier (section ``Out migration''), i.e., 20 cm for trout and 18 cm for salmon. The number of large (≥ 12 cm) out-migrants was not sensitive to the changes of the length at outmigration parameters (Fig. 34). This means using large out-migrants instead of total out-migrants was justified.

**Table 37:** Parameters for length at out-migration, mortFishOutmigrationL1 and mortFishOutmigrationL9, were varied by assuming fish stay longer in the G-Rapids compared to our initial assumption*.

Parameter	90% remain at					
	10 cm*		12 cm		14 cm	
	salmon	trout	salmon	trout	salmon	trout
mortFishOutmigrationL1	28.08	32.55	25.34	29.82	22.66	27.25
mortFishOutmigraitonL9	18.67	21.09	17.98	20.34	17.52	19.79

## Model output corroboration

As nearly all available data was used in parameterizing inSTREAM, we have not corroborated our inSTREAM predictions with independent data other than during validation of mean lengths (section ``Validation of predicted mean lengths of 0+ fish''). Our model application did however produce patterns of diel feeding and growth that future studies hopefully can corroborate.

### Diurnal and nocturnal feeding

**Fig. 35:**
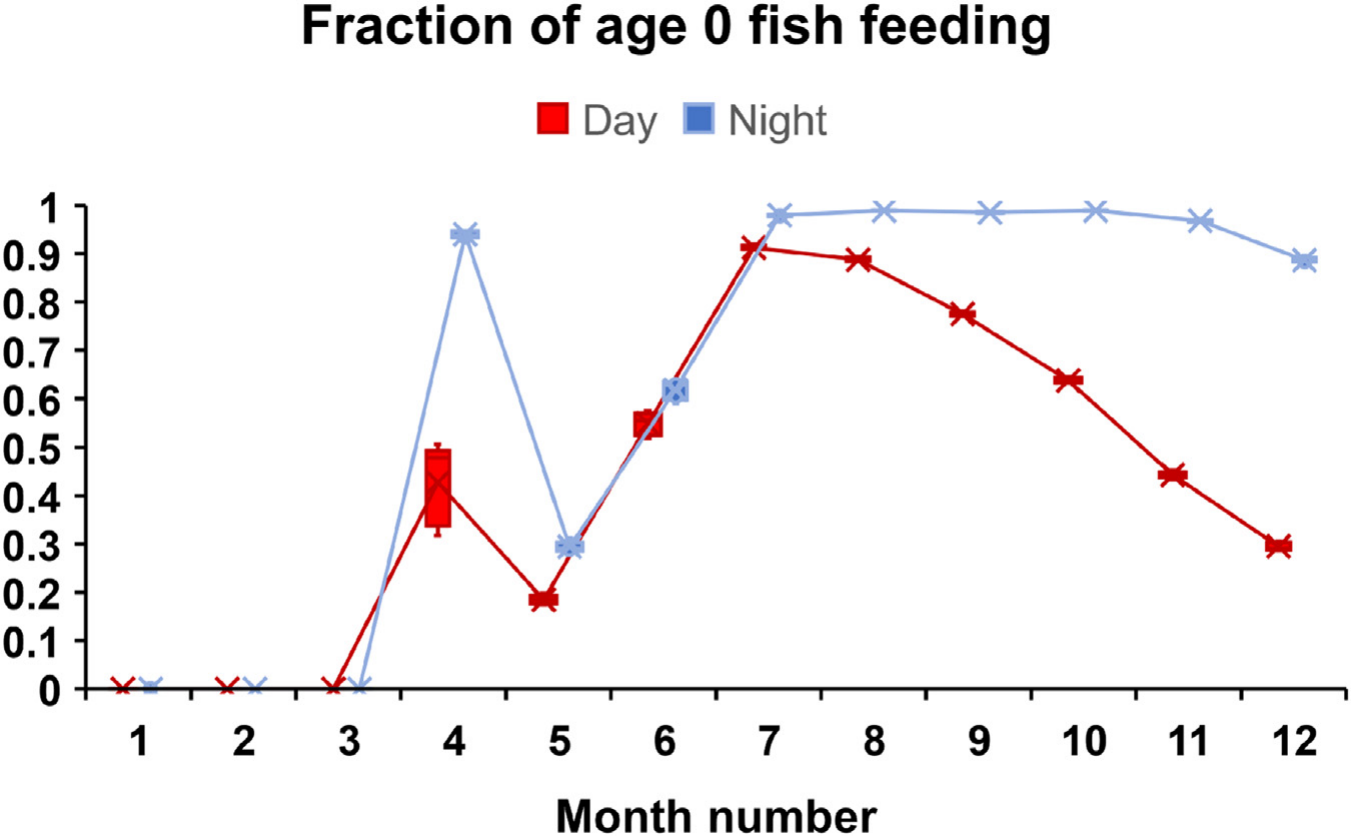
The average fraction of young of the year fish feeding during night and day over the calendar year.

**Fig. 36:**
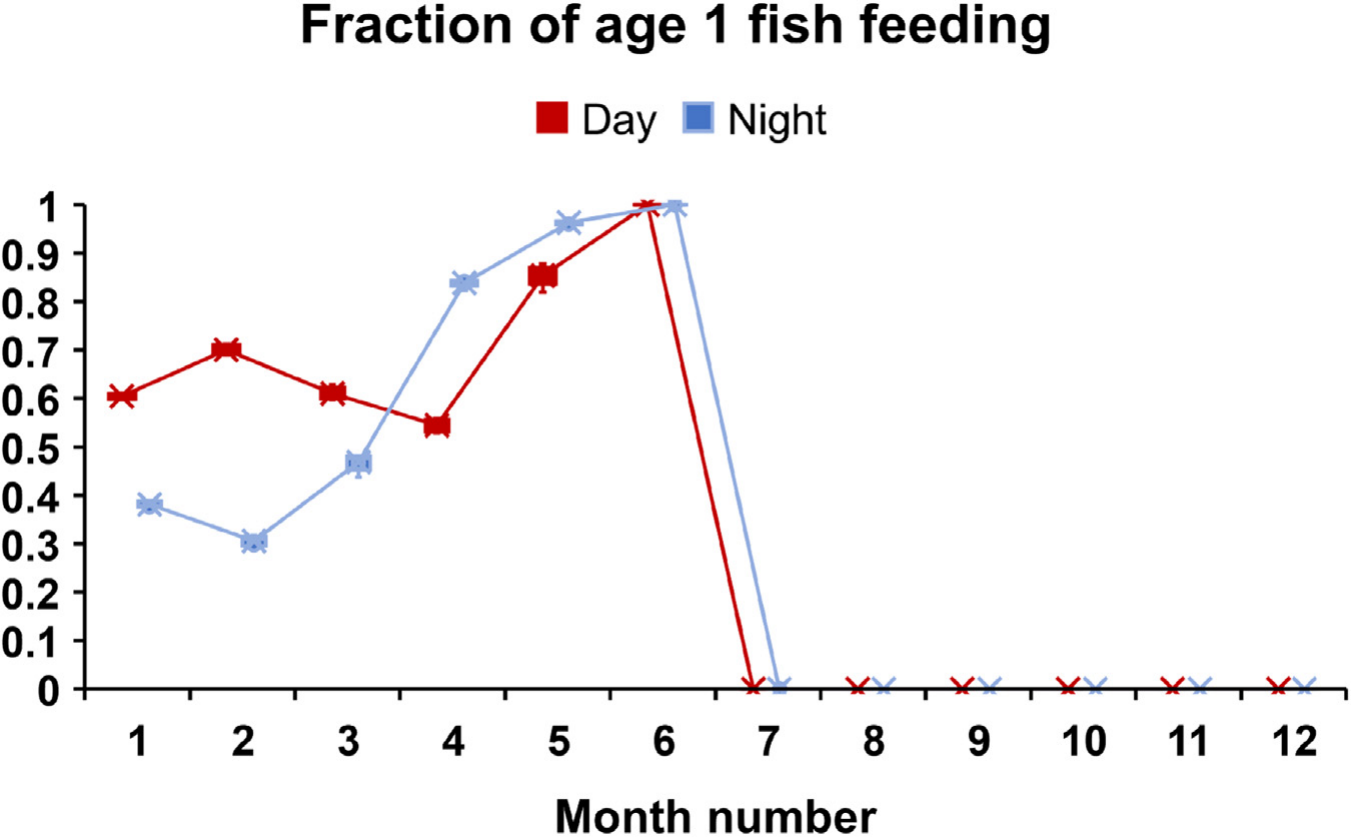
The fraction of age 1 fish feeding during night and day over the calendar year. By July, all 1+ fish have out-migrated.

We investigated the live fish output file for any differences in diurnal and nocturnal feeding patterns over the year. We used the R package dplyr [88] to aggregate the weighted (by number of fish) mean fraction of fish feeding at night (“phase”=0) and day (“phase”=1) per month, in replicates 1-5 of the null scenario for different age groups. Note that for the three of the winter months, January-March, young of the year (YOY) fish are still residing as eggs and alevins in the redd (Fig. 35). Likewise, inSTREAM predicts that all age 1 fish out-migrate before July (Fig. 36).

### Incremental growth of YOY fish

**Fig. 37:**
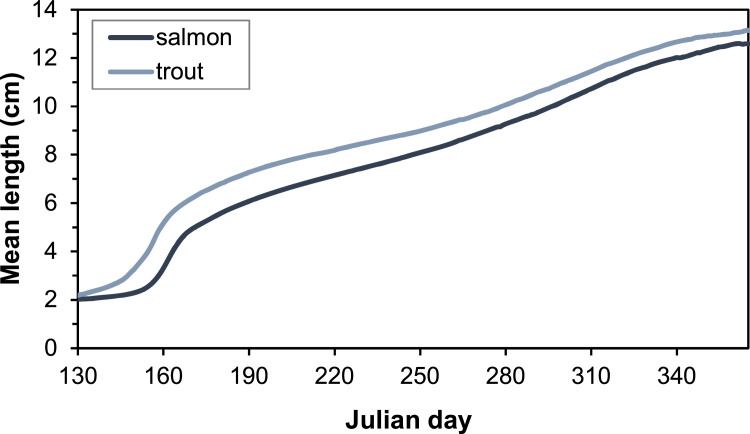
Illustration: mean length per Julian day of model years 2009-2018, replicate 1 of Flow0.

**Fig. 38:**
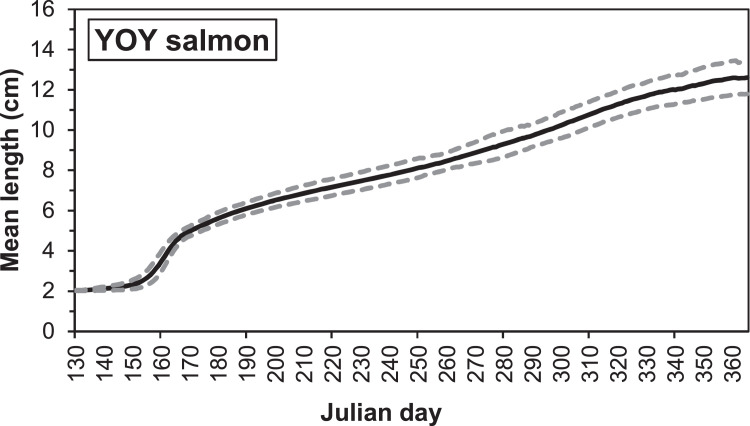
Simulated development in mean length of the YOY cohort of Atlantic salmon for model years 2009-2018 in replicate 1 of Flow0 averaged over Julian day (mean= black; mean ± SD = dotted). Julian day 130 corresponds to 10 May in a regular year.

**Fig. 39:**
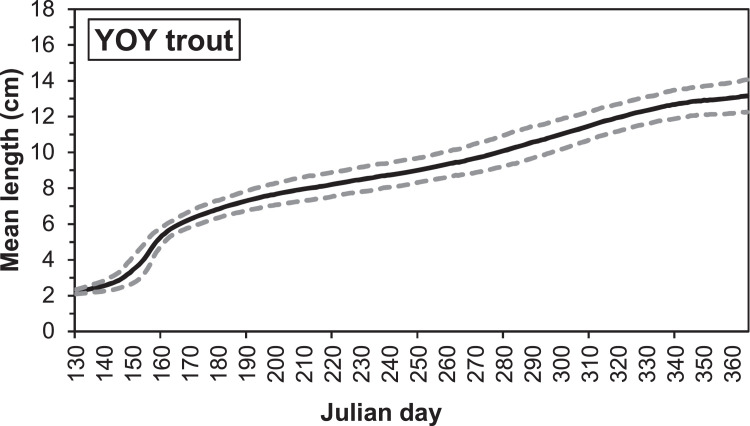
Simulated daily mean length of the YOY cohort of brown trout for model years 2009-2018 in replicate 1 of Flow0 averaged over Julian day (mean= black; mean ± SD = dotted). Julian day 130 corresponds to 10 May in a regular year.

We visually inspected the daily mean length of YOY fish, averaged over model years, from 10 May (Julian day 130) to 31 December (Julian day 365) for replicate 1 of the null scenario (Fig. 37, Fig. 38, Fig. 39). The average daily growth rate for salmon and trout was the same (0.043 (R^2^=0.95) and 0.045 (R^2^=0.97), respectively). The model predicts, correctly, that YOY brown trout on average are longer than YOY Atlantic salmon (Fig. 37). These growth curves could be tested if scale readings of juvenile or adult Gullspång salmon and trout are undertaken in coming years.

## Ethics statements

The electrofishing was done as part of an ongoing surveillance project in the Gullspång River by the river management group with a representative from the County Administrative Board as responsible manager. Electrofishing was done in accordance with Swedish and EU law. Handling of the fish was kept to a minimum and all fish were released alive back into the river after measurements were taken.

## Supplementary material and/or additional information [OPTIONAL]

Not applicable

## CRediT authorship contribution statement

**Kristine Lund Bjørnås:** Conceptualization, Validation, Formal analysis, Investigation, Writing – original draft, Visualization, Project administration. **Steven Railsback:** Methodology, Software, Validation, Resources, Data curation, Writing – review & editing, Supervision. **John Piccolo:** Conceptualization, Resources, Writing – review & editing, Supervision.

## Declaration of Competing Interest

The authors declare that they have no known competing financial interests or personal relationships that could have appeared to influence the work reported in this paper.

## Data Availability

Data will be made available on request.

## Footnotes

1 Database search parameters: Vattendrag = ”Gullspångsälven”, Lokal = ”Gullspångsforsen 1b”, ”1a”,”1c”, ”Turist” and ”2”.

2 SMHI:https://www.smhi.se/data/meteorologi/ladda-ner-meteorologiska-observationer/#param=airtemperatureInstant,stations=all,stationid=94050

3 Vattenwebb: www.miljodata.slu.se/mvm/Search
